# 14-3-3 Proteins and Other Candidates form Protein-Protein Interactions with the Cytosolic C-terminal End of SOS1 Affecting Its Transport Activity

**DOI:** 10.3390/ijms21093334

**Published:** 2020-05-08

**Authors:** Kerstin Duscha, Cristina Martins Rodrigues, Maria Müller, Ruth Wartenberg, Larry Fliegel, Joachim W. Deitmer, Martin Jung, Richard Zimmermann, H. Ekkehard Neuhaus

**Affiliations:** 1 Department of Plant Physiology, University of Kaiserslautern, Erwin-Schrödinger-Str., D-67653 Kaiserslautern, Germany; kerstin.duscha@gmx.de (K.D.); cmrodri@rhrk.uni-kl.de (C.M.R.); maria.e.mueller@googlemail.com (M.M.); wartenb@rhrk.uni-kl.de (R.W.); 2 Department of Biochemistry, Faculty of Medicine & Dentistry, University of Alberta, 347 Medical Sciences Building, Edmonton, AB T6G 2H7, Canada; lfliegel@ualberta.ca; 3 Department of Zoology, University of Kaiserslautern, Erwin-Schrödinger-Str., D-67653 Kaiserslautern, Germany; deitmer@biologie.uni-kl.de; 4 Department of Medical Biochemistry and Molecular Biology, Medical Faculty, Saarland University, D-66421 Homburg, Germany; martin.jung@uks.eu (M.J.); Richard.Zimmermann@uks.eu (R.Z.)

**Keywords:** Arabidopsis, salt tolerance, salt-overly sensitive (SOS1), 14-3-3 proteins, membrane transporter

## Abstract

The plasma membrane transporter SOS1 (SALT-OVERLY SENSITIVE1) is vital for plant survival under salt stress. SOS1 activity is tightly regulated, but little is known about the underlying mechanism. SOS1 contains a cytosolic, autoinhibitory C-terminal tail (abbreviated as SOS1 C-term), which is targeted by the protein kinase SOS2 to trigger its transport activity. Here, to identify additional binding proteins that regulate SOS1 activity, we synthesized the SOS1 C-term domain and used it as bait to probe *Arabidopsis thaliana* cell extracts. Several 14-3-3 proteins, which function in plant salt tolerance, specifically bound to and interacted with the SOS1 C-term. Compared to wild-type plants, when exposed to salt stress, Arabidopsis plants overexpressing SOS1 C-term showed improved salt tolerance, significantly reduced Na^+^ accumulation in leaves, reduced induction of the salt-responsive gene *WRKY25*, decreased soluble sugar, starch, and proline levels, less impaired inflorescence formation and increased biomass. It appears that overexpressing SOS1 C-term leads to the sequestration of inhibitory 14-3-3 proteins, allowing SOS1 to be more readily activated and leading to increased salt tolerance. We propose that the SOS1 C-term binds to previously unknown proteins such as 14-3-3 isoforms, thereby regulating salt tolerance. This finding uncovers another regulatory layer of the plant salt tolerance program.

## 1. Introduction

In nature, plants are constantly challenged by abiotic and biotic stress factors. Salinization is an increasingly common abiotic stress, leading to reduced growth, impaired development, and significantly reduced crop yields [1]. Since sodium chloride (NaCl) is present in nearly all types of soil, flowering plants have developed a sophisticated multifactorial strategy to withstand situations in which salt is present in excess. This strategy can be sub-categorized into at least three modes, namely (i) the avoidance of salt uptake, (ii) Na^+^ export from the cell across the plasma membrane, and (iii) internal cellular compartmentalization into the central vacuole [2,3,4].

Plants have various morphological and structural features that limit Na^+^ uptake into the plant body, and the intracellular sequestration of salt is catalyzed by electroneutral Na^+^/H^+^ antiporters of the NHX (Na^+^/H^+^ Exchanger) protein family. In *Arabidopsis thaliana*, the NHX family comprises eight isoforms, only two (NHX7 and NHX8) of which reside in the plasma membrane [3,5]. NHX7 is also referred to as SALT-OVERLY-SENSITIVE1 (SOS1) because *SOS1* was first identified in a forward-genetic screen for Arabidopsis mutants with compromised salt-stress tolerance [3,6]. Subsequent studies revealed that the SOS system of flowering plants consists of three components: the Na^+^/H^+^ exchanger SOS1; the protein kinase SOS2, which activates SOS1; and SOS3, a plasma membrane-located calcium sensor that induces SOS2 activity upon the onset of salt stress [7].

Due to the proton-motive force across the plasma membrane, the highly active SOS1 transporter pumps Na^+^ permanently out of the cell, which is beneficial under conditions of excess soil salinity. The observation that overexpressing *SOS1* confers salt tolerance in both the model plant Arabidopsis and in crop species such as tomato (*Solanum lycopersicum*) highlights the importance of this transporter in salt tolerance in higher plants [8,9]. Some plant species contain low levels of sodium ions, which are required for proper chloroplast function [10,11], suggesting that a minimum Na^+^ concentration must be maintained in plant cells. Interestingly, *sos1* loss-of-function mutants not only exhibit increased salt sensitivity, but they also show impaired potassium homeostasis [6,12]. Therefore, SOS1 activity must be stimulated to ensure increased sodium export under salt stress, while SOS1 activity must be reduced to inhibit sodium export in the presence of an optimal salt supply.

The dynamic modulation of SOS1 activity is achieved by various processes such as altered gene expression, modified mRNA stability, and post-translational modification via reversible phosphorylation [13,14]. *SOS1* expression in Arabidopsis is induced by salt treatment [15], suggesting that salt might induce *SOS1* transcript stabilization [16], as well as increased *SOS1* mRNA levels. SOS1 is specifically targeted by the protein kinase SOS2. In turn, SOS2 activity is stimulated by the plasma membrane-bound calcium sensor SOS3 in response to salt stress [17]. Several independent observations suggest that SOS2 is a critical element of the plant salt tolerance program: (i) SOS2 is recruited to the plasma membrane where it phosphorylates SOS1, which triggers Na^+^/H^+^ exchange by this transporter [18]; (ii) SOS2 interacts with the calcium sensor CBL10, resulting in the association of this protein complex with the tonoplast to stimulate vacuolar sodium accumulation [19]; and (iii) sequestration of SOS2 by the regulatory 14-3-3 proteins or by the flowering regulator protein GIGANTEA represses the SOS pathway [20,21]. Indeed, the concerted interaction of all three SOS elements allowed the complete Arabidopsis pathway to be functionally reconstituted in yeast cells [22]. The absence of only a single SOS component substantially increases salt sensitivity [23].

SOS2-dependent phosphorylation of SOS1 takes place on the markedly long (~700 amino acids) cytosolic C-terminal hydrophilic extension of this transporter. In the absence of phosphorylation, this C-terminal end (hereafter referred to as C-term) acts as an autoinhibitory domain that maintains SOS1 in a low-activity resting state [24]. Interestingly, the C-terminal domain appears to be conserved in all plant plasma membrane Na^+^/H^+^ antiporters and is also functional in the human Na^+^/H^+^ Exchanger isoform1 (NHE1) [25]. NHE1 is a ubiquitous carrier in the plasma membranes of animal cells that mainly functions in pH regulation by expelling a proton while simultaneously importing an extracellular Na^+^ ion; this transport direction is the opposite to that of SOS1.

The C-terminal end of NHE1 is approximately 315 amino acids long and harbors multiple protein phosphorylation sites. To date, 30 proteins have be shown to bind to the C-terminus of NHE1, which is structurally similar to the corresponding domain in SOS1, including an impressively large number of regulatory factors. Among these NHE1-binding partners are several protein kinases, protein phosphatases, calmodulin, a calcineurin homologous protein, various 14-3-3 proteins, proteins that facilitate interactions of NHE1 with the cytoskeleton, and small signaling molecules such as phosphatidylinositol 4,5-bisphosphate [26]. This plethora of NHE1 effector molecules implies that its regulatory mechanisms are complex, allowing for the sophisticated fine-tuning of this carrier based on dynamic cellular demands.

Thus far, only a few protein–protein binding partners have been identified for the C-term domain of SOS1, including the protein kinase SOS2; RCD1, which regulates the oxidative-stress response; and the mitogen-activated protein kinase MAPK6 [27,28]. Therefore, we hypothesized that the large C-term domain of SOS1, which is twice the size of the already large C-terminal domain of NHE1, might harbor unidentified binding sites for regulatory protein–protein interactions.

Here, we employed three major strategies to test this hypothesis: (1) we used recombinant SOS1 C-term protein as bait to identify novel binding proteins in Arabidopsis cell extracts; (2) we verified candidate protein–protein interactions by bimolecular fluorescence complementation (BiFC) and peptide-spot analyses; and (3) we overexpressed the hydrophilic C-terminal domain of SOS1 in planta and analyzed the salt-stress response in the corresponding transformants. The overexpression of the soluble portions of hydrophobic transport proteins in either living cells or as bait molecules has been successfully used to identify binding proteins under native conditions or in vitro [29,30,31]. Our results provide compelling evidence that the SOS1 C-term domain binds to several previously unknown proteins. Among these are 14-3-3 proteins, which are thought to participate in salt-stress tolerance in plants and thus appear to contribute to the fine-tuning of SOS1 activity in vivo.

## 2. Results

### 2.1. Identification of Proteins Able to Bind to the C-Term Part of SALT-OVERLY-SENSITIVE1 (SOS1)

**Figure 1:**
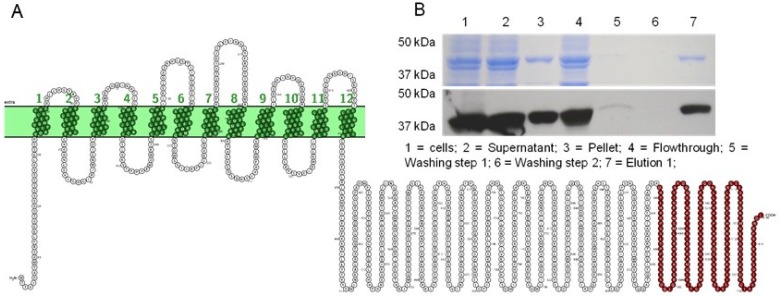
Expression and purification of the recombinant *At*SOS1 C-terminus (aa978–1146). (**A**) Model of the SALT-OVERLY-SENSITIVE1 (SOS1) antiporter; the target region used for the pull-down assay is marked in red. (**B**) SDS–PAGE and Western blot of purified *At*SOS1 C-terminus (aa978–1146). Glutathione-sepharose chromatography was performed using the GST-tagged fusion protein. 1: total proteins from *At*SOS1 C-terminus (aa978–1146) expressing *E. coli* cells; 2: supernatant (after centrifugation at 10,000× *g*); 3: cell pellet (after centrifugation at 10,000× *g*); 4: flowthrough; 5: washing step 1; 6: washing step 2; 7: elution 1 (apparent molecular mass was 44 kDa). The molecular mass (kDa) of the marker proteins is indicated.

As described in the introduction, we posit that the large hydrophilic C-term portion of SOS1 is able to bind additional regulatory proteins than identified thus far. To explore putative binding proteins able to interact with this SOS1 domain we exploited a method which already allowed the identification of regulatory proteins interacting with a hydrophilic domain of a transporter protein [30]. To this end we expressed the C-terminal domain of SOS1, corresponding to the amino-acid positions 978 to 1146 (Figure 1A), as a Glutathione-S-Transferase-(GST) tagged fusion protein in *Escherichia coli* and purified the recombinant protein to an apparent homogeneity via glutathione-sepharose chromatography (Figure 1B, lane 7). Subsequently, the purified recombinant protein of an apparent molecular mass of 44kDa (Figure 1B) was concentrated and coupled to an activated Affi-gel10 affinity matrix. The coupled protein was incubated with a soluble protein extract prepared from Arabidopsis leaves to interact with putative binding partners. (note: for unknown reasons we were not able to express larger SOS1 C-term constructs in *E. coli*).

After allowing the interaction of soluble leaf proteins with the SOS1 C-term, non-specifically bound Arabidopsis proteins were removed by washing the column with phosphate-buffered saline (PBS). Hereafter, specifically bound proteins were dissociated from the C-term protein by elution with PBS containing 1% sodium-dodecyl sulfate, recovered, and separated via SDS-PAGE electrophoresis (Figure S1, lane 1). This protein lane was cut in slices and proteins were identified by mass spectrometry. To identify Arabidopsis proteins that bind non-specifically to GST peptides, the same treatment was carried out with GST protein expressed and purified from *E. coli* (Figure S1, lane 2).

Mass spectrometry (mass spec.) allows for highly sensitive detection and identification of proteins. Using mass spec.,143 individual proteins were identified in the eluate of the SOS1 C-term specifically bound partners and were ranked according to their relative abundance (Table S1). Apart from various tubulin isoforms, five different 14-3-3 like protein isoforms appeared within the first 15 most abundant proteins physically associated with the C-terminal SOS1 protein (Suppl. Table S1). The 14-3-3 isoforms were *omega* (*ω*), *phi* (*φ*φ {\displaystyle \varphi \,}), *kappa* (*κ*), *upsilon* (*υ*) and *nu* (*ν*). Latter proteins gained our special attention, since binding of 14:3:3 isoforms to the sodium/proton exchanger NHE, representing a human homolog to SOS1, modulates its activity [26]. In addition to these Arabidopsis 14:3:3 isoforms, a number of metabolic enzymes were among the 15 most abundant interacting proteins: *inter alia* adenosyl-homocysteinease2, enolase2, delta-1-pyrroline-5-carboxylate synthase (P5CS) and S-adenosylmethionine synthase1 (Table S1). Interestingly both, adenosyl-homocysteinease and delta-1-pyrroline-5-carboxylate synthase represent enzymes known be of relevance for plant osmoregulation (see Table S1). 

### 2.2. Investigating the Physical Interaction of 14-3-3 Isoforms and the C-Terminal end of SOS1 in the Living Cell

To verify if the 14-3-3 isoforms interaction was a specific physical interaction with the recombinant C-terminal end of SOS1 in vivo, bimolecular fluorescence complementation (BiFC; [32]) was employed. The method is based on the association of complementary yellow fluorescent protein (YFP) fragments fused to the potential partner proteins. Close proximity of the complementary fragments results in the (re)formation of the fluorescing marker protein.

We focused on the 14-3-3 isoforms upsilon, omega, kappa and lambda and transformed Nicotiana leaf epidermis cells accordingly. Because the 14-3-3 *lambda* isoform was shown earlier to be involved in the regulation of the SOS pathway [21] we supplemented our set of putative binding partners for BiFC studies with the *lambda* isoform. Accordingly, the following plasmid combinations were infiltrated into epidermal cells: *C-term::yfp^NT^* and *14-3-3ω::yfp^CT^*, *C-term::yfp^NT^* and *14-3-3υ::yfp^CT^*, *C-term::yfp^NT^* and *14-3-3κ::yfp^CT^* as well as *C-term::yfp^NT^* and *14-3-3λ::yfp^CT^*. To confirm that recombinant *C-term::yfp^NT^* does not interact non-specifically with every other ::yfp^CT^ protein we also transformed Nicotiana cells with *C-term::yfp^NT^* and *vik::yfp^CT^* as a negative control (Figure S2).

**Figure 2:**
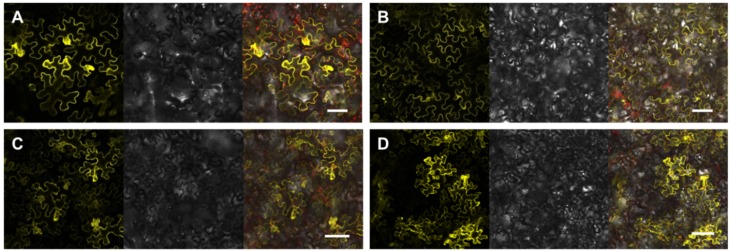
Bimolecular fluorescence complementation analysis in *N. benthamiana*. Plasmids containing the generated *yfp_NT_* and *yfp^CT^* constructs were transiently co-transformed into *N. benthamiana* leaves using *A. tumefaciens* mediated transformation. Left image: YFP fluorescence, middle image: transmitted light and right image: merge of YFP fluorescence, chlorophyll fluorescence and transmitted light. The YFP fluorescence signal was detected using a Leica TCS SP5II confocal laser scanning microscope system. Bar = 100 µm. (**A**) *14-3-3* *υ::yfp^CTT^* and *sos1C-term::yfp^NT^.* (**B**) *14-3-3* *ω::yfp^CT^* and *sos1C-term::yfp^NT^.* (**C**) *14-3-3* *κ::yfp^CT^* and *sos1C-term::yfp^NT^.* (**D**) *14-3-3* *λ::yfp^CT^* and *sos1C-term::yfp^NT^.*

Co-expression of *C-term::yfp^NT^* with either *14-3-3υ::yfp^CT^*, *14-3-3ω::yfp^CT^*, *14-3-3κ::yfp^CT^* or *14-3-3λ::yfp^CT^* always resulted in a clearly visible BiFC signal in epidermal cells (Figure 2A–D). These defined signals localized to the cytosol and varied in fluorescence intensity. To validate the BiFC data, a control experiment was conducted with an alternative partner protein, the protein kinase VIK1. Although VIK1 is generally expressed as a *vik::yfp^CT^* fusion protein [30], no interaction with the SOS1 C-term fusion protein was detectable (Figure S2).

### 2.3. Identification of the Binding Site for 14-3-3ω on the SOS1 C-Term Protein

To identify the SOS1 C-term binding sites able to interact with 14-3-3 proteins we conducted a peptide-spot analysis [33]. For this analysis, 15-mer synthetic peptides that sequentially overlap by 12 amino-acid residues, representing an extended version of the C-terminal extension of the SOS1 transporter starting from amino acid 446, were spotted on a cellulose membrane (Figure 1A and Table S2).

**Figure 3:**
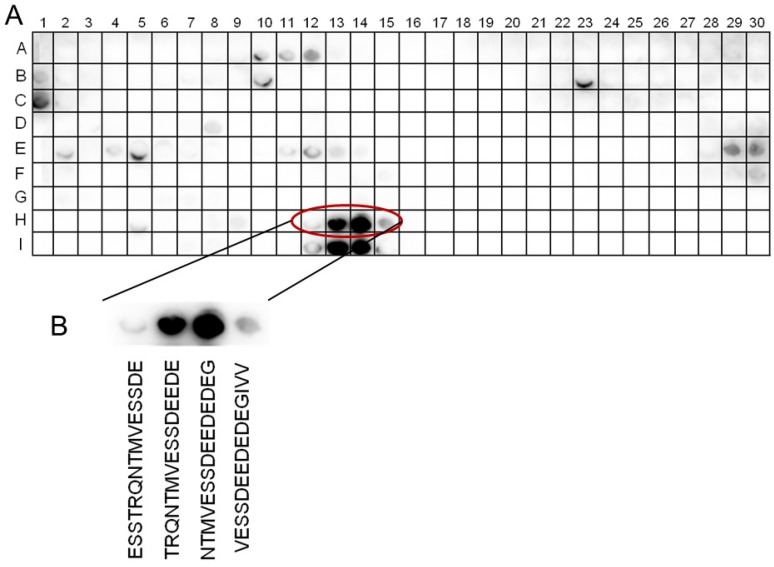
Peptide-spot binding assay with *At*SOS1 C-terminus. Fifteen amino acid long peptides were spotted on a cellulose membrane and incubated with recombinant (10xHis)-labelled *At*14-3-3ω (**A**). The spots correspond to the respective region of *At*SOS1 C-terminus as indicated. (**B**) Anti-poly-Histidine-antibody in combination with an HRP-coupled anti-mouse antibody, ECL™ and luminescence imaging were employed using an Odyssey^®^ Fc Imaging System (LI-COR Biosciences, Lincoln, NE, USA).

The membrane-bound peptides were incubated with recombinantly synthesized His-tagged 14-3-3*ω* protein (see Figure S3 lane 2; as a representative 14-3-3 isoform able to interact with the C-term protein under in vivo conditions, see Figure 2B). The interaction between 14-3-3*ω* and individual SOS1 C-term peptides was visualized by means of an anti-His antibody. The binding of 14-3-3*ω* was detected at several domains of this C-terminal SOS1 protein with low efficiency. However, binding of 14-3-3*ω* to the C-terminal protein led to a remarkably strong staining of two successive peptide spots, spanning the amino-acid positions 1112 to 1129 (spot numbers H13 and H14, Figure 3A). These two spots cover the amino-acid sequence ^1112^TRQNTMVESSDEEDEDEG^1129^ of the SOS1 C-terminal end (Figure 3B).

Typically, 14-3-3 type proteins bind to phosphorylated amino-acid residues in conserved domains [34] and in the case of SOS1, the amino-acid residue Ser_1138_ represents the target of the protein kinase SOS2 [24]. Thus, it was interesting to see whether a phospho-mimicry mutation at this position might improve 14-3-3*ω* binding. To test this, the spots with number H5–H20 in the second lower lane of peptides were spotted in a replica with one change at amino acid serine 1138 (spot numbers I17-I20). This was either present as the native peptide spots with the sequence *DEDEGIVVRIDSP**S**_1138_KIVFRNDL* (upper lane), or as a site directed phospho-mimicry changing Ser_1138_ for a negatively charged aspartate residue, leading to the peptide spot sequence *DEDEGIVVRIDSP**D**_1138_KIVFRNDL*. As visualized, in neither case was there evidence of a significant interaction between 14-3-3*ω* and the bait peptide (Figure 3A).

### 2.4. Design of Arabidopsis p35s::SOS1^c-term^StrepII Mutants Overexpressing the C-Terminal End of SOS1

**Figure 4:**
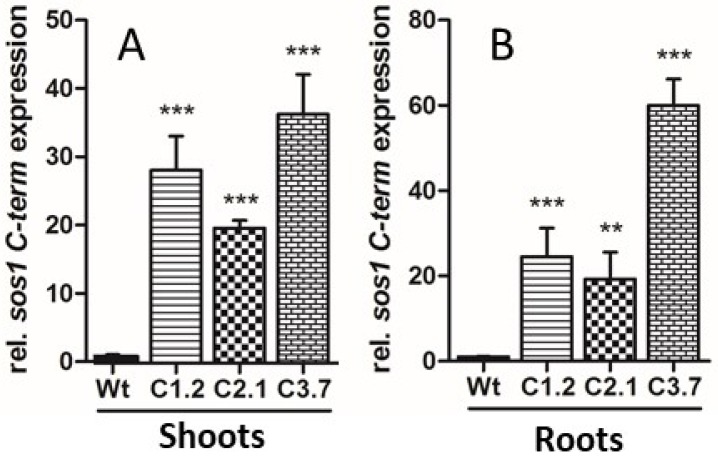
Relative overexpression levels of *SOS1-C-terminus* in Arabidopsis shoot and root tissue, revealed by quantitative RT-PCR. (**A**) Relative overexpression of *SOS1-C-terminus* in shoots, (**B**) relative overexpression of *SOS1-C-terminus* in roots. Arabidopsis plants were grown in hydroponic culture for five weeks, prior to watering for three days with 150 mM NaCl. Data represent means of six independent biological replicates. Data normalized to the housekeeping gene *pp2a* (*At1g13320*). Error bars are ±SE. Data with *** are significant from Wt at *p* < 0.001, data with ** are significant from Wt at *p* < 0.01 (two-way ANOVA).

Given the growing evidence that the C-terminal portion of SOS1 binds additional regulatory proteins than known before, we were interested to reveal whether increased levels of the protein fragment would alter plant properties to respond to salt stress. To this end, we cloned a cDNA coding for amino acids 446 to 1146 of the SOS1 C-terminal protein, representing the hydrophilic sequence extension of the transporter, 5′ upstream of a *Strep-*tag II fusion [24], leading to *p35s::SOS1^c-term^StrepII* plants. After the transformation of wild type Arabidopsis plants, we identified several antibiotic resistant mutant lines and focused on three individual *p35s::SOS1^c-term^StrepII* lines (designated C1.2, C2.1 and C3.7). All three mutant lines exhibited markedly increased *C-term* mRNA levels in leaves (shoots) and in roots, when compared to corresponding Wt controls (Figure 4A,B).

To confirm increased levels of the SOS1 C-term fragment in transgenic plants we prepared whole protein extracts from root tissue of salt stressed Wt- or *p35s::SOS1^c-term^StrepII* plants. For the detection of overexpressed proteins in *p35s::SOS1^c-term^StrepII* mutants, we took advantage of the *Strep-*tag II. Since the level of C-term protein in the extracts remained relatively low, we conducted dot-blot assays on a nitrocellulose membrane using a *Strep*-tag II antibody on spotted root extracts. In all three *p35s::SOS1^c-term^StrepII* lines we observed increased levels of the corresponding protein over background immunoreactivity, confirming that SOS1 C-term mRNA corresponds to C-term protein (Figure S4).

To check for the putative presence of an unintended co-suppression phenomenon, caused by high levels of *SOS1* mRNA in *p35s::SOS1^c-term^StrepII* mutants, we also quantified the authentic *SOS1* mRNA in shoots and roots, respectively (Figure S5). No significant alteration in endogenous *SOS1* mRNA level was detected confirming that unwanted co-suppression did not occur (Figure S5).

### 2.5. Onset of Flowering in p35s::SOS1^c-term^StrepII Plants Is Less Impaired by High Salt Concentrations When Compared to Wild Types

**Figure 5:**
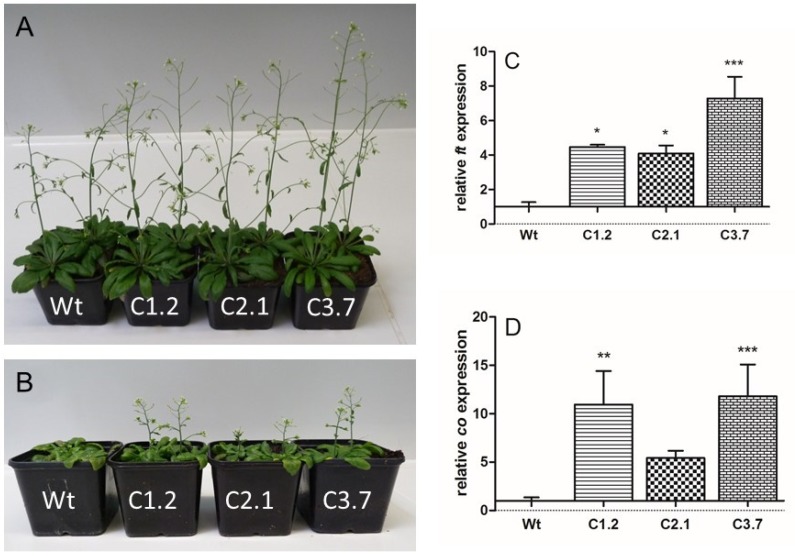
Analysis of plant development on soil of Wt and *p35s::SOS1^c-term^StrepII* mutants. Plants were grown on soil for three weeks prior to transfer to long-day conditions. For the following two weeks, plants were treated with the same volume of either tap water (**A**) or tap water containing 150 mM NaCl (**B**) every three days. Pictures were taken after seven weeks of growth. (**C**) Relative transcript levels of *Flowering Locus T* in Arabidopsis leaves under salt stress conditions. (**D**) Relative transcript levels of *CONSTANS* in Arabidopsis leaves under salt stress conditions. Data represent means of three independent biological replicates, +/- SE. Data with *** are significant from Wt at *p* < 0.001, data with ** are significant from Wt at *p* < 0.01 and data with * are significant from Wt at *p* < 0.05 (two-way ANOVA) Data are normalized to the housekeeping gene *pp2a* (*At1g13320*).

Within the first seven weeks of growth on soil we did not observe substantial alterations of plant development between *p35s::SOS1^c-term^StrepII* lines and wild type. All plants developed similarly and exhibited well-established inflorescences (Figure 5A). Interestingly, substantial differences between the three *p35s::SOS1^c-term^StrepII* lines and wild type controls emerged if plants were grown under soil salinity (150 mM NaCl). Flower development was obvious and found to be significantly compromised in all four plants lines. However, in contrast to wild type plants, in which onset of inflorescence formation was entirely prevented (Figure 5B), floral development in all *p35s::SOS1^c-term^StrepII* mutants was less severely affected as indicated by frequent inflorescence formation during the analyzed time span (Figure 5B).

Flowering of higher plants is a complex process governed by various molecular and physiological alterations, and clearly delayed upon salt stress [35]. To check whether salt stress differentially affects the expression of the flowering genes *FT* (Flowering Locus T) or *CONSTANS* in wild types and *p35s::SOS1^c-term^StrepII* mutants, we quantified the corresponding mRNA levels via qRT-PCR. All three *p35s::SOS1^c-term^StrepII* mutants contained higher levels of *FT* mRNA when compared to wild types (Figure 5C). Similarly, *CONSTANS* mRNA in C-term lines C1.2 and C3.7 is significantly higher when compared to *CONSTANS* mRNA levels found in wild type plants (Figure 5D). These findings indicate that induction of flowering is less impaired in *p35s::SOS1^c-term^StrepII* mutants under conditions of salt stress, when compared to corresponding wild type plants.

### 2.6. C-Term Mutants Are Less Affected by High Salt Concentrations and Contained Less Sodium than Wild Type

**Figure 6:**
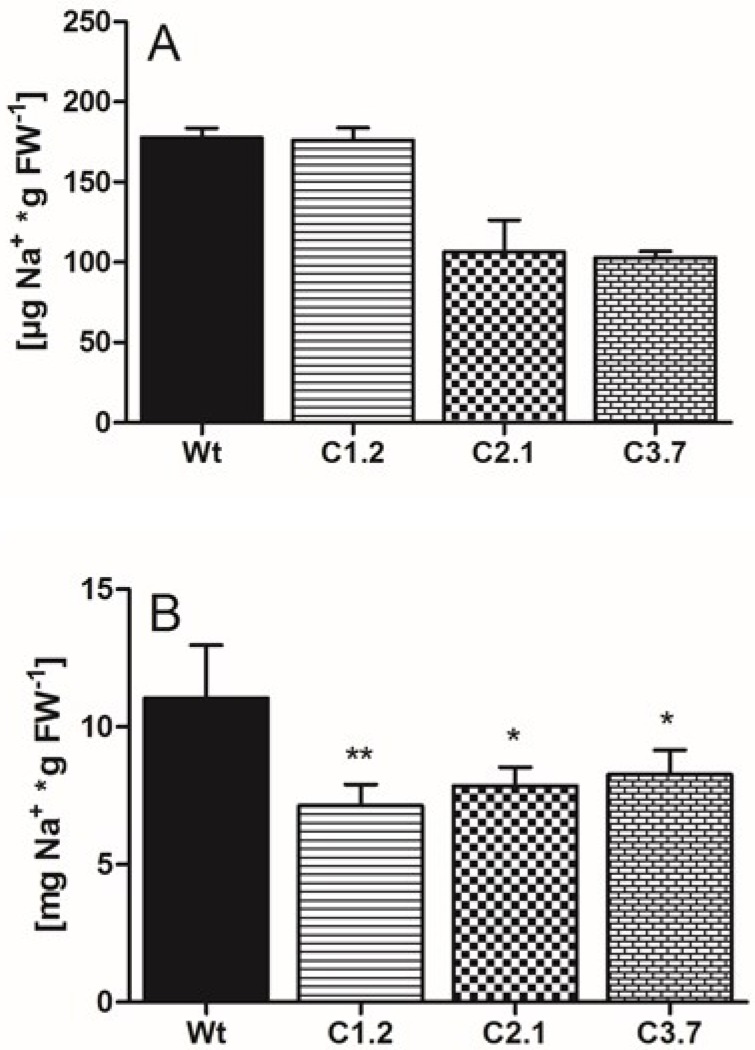
Sodium content in Wt and *p35s::SOS1^c-term^StrepII* plants. Wt and *p35s::SOS1^c-term^StrepII* plants were grown on soil for three weeks prior to transfer to long-day conditions. For the following two weeks, plants were treated with the same volume of either 150 mM NaCl solution or tap water every three days. Samples for ion quantification were taken after an additional two weeks. Na^+^ concentrations in leaves under control (**A**) and under salt stress conditions (**B**), quantified by Ion chromatography. Values represent the mean of 3 biological replicates, each with 2 technical replicates. Error bars are ±SE. Data with *, ** are significant from Wt at *p* < 0.05 or *p* < 0.01 (two-way ANOVA).

To check for a correlation between altered flowering and sodium levels in *p35s::SOS1^c-term^StrepII* mutants we quantified leaf Na^+^ levels in soil grown mutants and wild types. In the absence of additional salt in the soil, Na^+^ levels in all plant lines were markedly low and ranged below 180 µg_*_gFw^−1^ (Figure 6A). As expected, addition of sodium chloride to the water led to a substantial increase in leaf sodium levels in all four plant lines. Wild type leaves contained about 11 mg Na^+^_*_gFw^−1^, while leaves from the three *p35s::SOS1^c-term^StrepII* lines exhibited significantly less sodium, ranging from 7.1 mg Na^+^_*_gFw^−11^ in line C1.2 to 8.3 mg Na^+^_*_gFw^−1^ in line C3.7 (Figure 6B).

**Figure 7:**
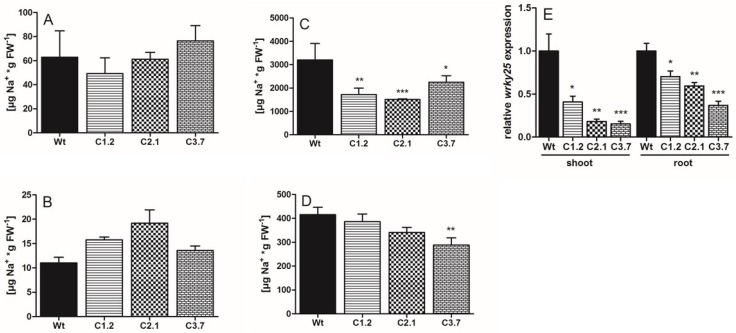
Sodium content and relative expression levels of the salt-stress indicator gene *wrky25* in Wt and *p35s::SOS1^c-term^StrepII* plants. Wt and *p35s::SOS1^c-term^StrepII* plants were grown in hydroponic culture for five weeks and watered with ± 100 mM NaCl 72 h prior to measurement. Na_+_ concentrations in leaves (**A**) and roots (**B**) under control and under salt stress conditions (leaves **C**, roots **D**), quantified by IC. Relative transcript levels of *wrky25* in Arabidopsis shoot and root tissues under salt stress conditions (**E**). Data are normalized to the housekeeping gene *pp2a* (*At1g13320*). Values represent the mean of 3 biological replicates, each with 2 technical replicates. Error bars are ± SE. Data with *, **, *** are significant from Wt at *p* < 0.05, *p* < 0.01 or *p* < 0.001 (two-way ANOVA).

Plant growth in pots does not allow harvesting of root tissue without soil contamination. Thus, to compare sodium levels in shoots and in roots, we grew Arabidopsis in hydroponics. To this end, we solubilized corresponding plant tissues and quantified subsequently Na^+^ therein. When grown under control conditions both, shoots and roots of all four plants lines contained similar levels of Na^+^ amounting to about 63 µg_*_gFw^−1^ in shoots, and between 11 and 19 µg Na^+^_*_gFw^−1^ in roots, respectively (Figure 7A,B). After addition of salt to the hydroponic growth medium, Na^+^ in wild type shoots increased substantially and reached about 3200 µg_*_gFw^−1^ (Figure 7C). In contrast, Na^+^ levels in *p35s::SOS1^c-term^StrepII* lines were significantly lower and amounted only to 1510 µg_*_gFw^−11^ in line C2.1 and 2250 µg_*_gFw^−1^ in line C3.7 (Figure 7C). Na^+^ levels in wild type roots also increased by the additional presence of salt in the growth medium and reached 415 µg_*_gFw^−1^ (Figure 7D). Na^+^ levels in root tissue from *p35s::SOS1^c-term^StrepII* lines C1.2 and C2.1 appeared to be comparable to the level observed in wild types, while Na^+^ in *p35s::SOS1^c-term^StrepII* line C3.7 was slightly lower than observed in wild type tissues and amounted to 288 µg/gFw_*_gFw^−1^ (Figure 7D).

It is well-known that Na^+^ accumulation in plant cells triggers a variety of physiological responses on different levels including metabolism, post-translational protein modifications, and gene expression [36]. Among the salt-induced genes are a plethora of transcription factors, such as *WRKY25* that plays a critical role in activating salt tolerance in higher plants (see e.g., [37]). Therefore, we quantified *WRKY25* mRNA levels after salt treatment in both shoots and roots of *p35s::SOS1^c-term^StrepII* mutants compared to wild-type controls. We found that *WRKY25* transcripts were significantly lower in all three *p35s::SOS1^c-term^StrepII* mutants than in correspondingly treated wild type plants (Figure 7E).

To check whether C-term plants show any metabolic alteration when compared to wild type plants we monitored the concentration of various sugars, starch, and the amino acid proline. Soluble sugars and proline represent metabolites exerting a protective function during salt- and osmotic stress. Additionally, total starch levels correlate positively with tolerance against salt stress [36,38,39].

**Figure 8:**
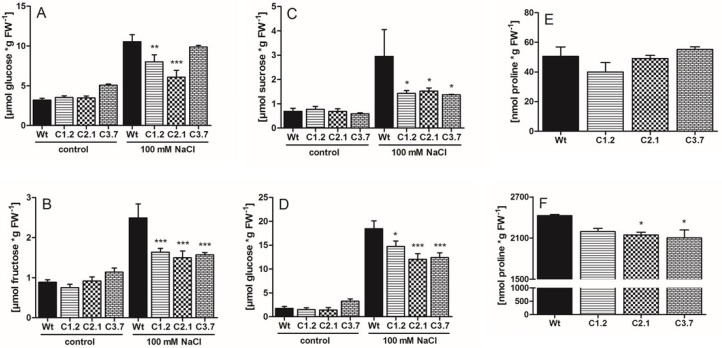
Metabolite accumulation in Arabidopsis leaves during 72 h of salt stress. (**A**) glucose, (**B**) fructose, (**C**) sucrose, (**D**) starch (in glucose equivalents), (**E**,**F**) proline. Wt plants and *35S::SOS1 C-terminus* overexpression lines were grown in hydroponic culture for five weeks and watered ±100 mM NaCl 72 h prior to measurement. Sugar accumulation was determined in leaves under control and under salt stress conditions, quantified by IC. Proline accumulation was quantified by HPLC. Values represent the mean of 3 biological replicates, each with 2 technical replicates. Error bars are ±SE. Data with *, **, *** are significant from Wt at *p* < 0.05, *p* < 0.01 or *p* < 0.001 (two-way ANOVA).

When grown under hydroponic control conditions, wild type and all *p35s::SOS1^c-term^StrepII* lines were not significantly different containing between 3.2 and 5.1 µmol Glc_*_gFw^−1^ (Figure 8A). After exposure to salt stress, Glc levels in all four plant lines increased substantially with up to 10.5 µmol_*_gFw^−1^ in wild type plants. However, in *p35s::SOS1^c-term^StrepII* lines C1.2 and C2.1 salt stress-induced glucose accumulations were less pronounced and reached only 8.0 and 6.1 µmolGlc_*_gFw^−1^ in lines C1.2 and C2.1, respectively, while Glc levels in the mutant line C3.7 even slightly decreased (Figure 8A). Similar trends were found for fructose in all four plant lines after exposure to salt stress. In the case of wild type plants, fructose increased about 2.8-fold, namely from 0.89 to 2.5 µmol_*_gFw^−1^, whereas all *p35s::SOS1^c-term^StrepII* lines remained low fructose levels when compared to wild types, with a maximum of only 1.64 µmol_*_gFw^−1^ in line C1.2 (Figure 8B). Under control conditions all four plant lines contained about 0.68 µmol sucrose_*_gFw^−1^ (Figure 8). Similar to Glc and Fru changes, salt stress-induced sucrose accumulation was most pronounced in wild type leaves (about 4.3-fold), while all three *p35s::SOS1^c-term^StrepII* lines exhibited much less increased sucrose after salt stress (about 2-fold increase, Figure 8C). As seen for sugars, starch levels in wild type leaves rose substantially after salt stress, namely from 2 µmolC6_*_gFw^−1^ under control conditions to about 18.5 µmolC6_*_gFw^−1^ upon salt stress (Figure 8D). In contrast, starch in the three *p35s::SOS1^c-term^StrepII* lines C1.2, C2.1 and C3.7 was significantly less increased when compared to wild type plants, and reached only 14.7, 12.1, and 12.4 µmolC6_*_gFw^−1^, respectively (Figure 8D).

Under control conditions, proline levels in leaves from both, wild-type and C-term plants appeared to be similar and ranged, without statistical difference, between 40 and 55 nmol_*_gFw^−1^ (Figure 8E). As expected, after salt stress, proline accumulated substantially in all four plant lines and reached 2430 nmol_*_gFw^−1^ in wild type plants (Figure 8F). However, the three C-term mutants contained less proline than present in corresponding wild type leaves (Figure 8F).

## 3. Discussion

Vascular plants have developed a sophisticated system of morphological, physiological, and molecular responses that allow them to tolerate saline soil conditions [36]. The plasma membrane transporter SOS1 plays a critical role in these responses by exporting excess Na^+^ in counter-exchange with protons. Thus, SOS1 not only reduces cytosolic sodium levels, but it also removes it completely from the cell.

The structure of SOS1 is remarkable, as this transporter contains an extremely long C-terminal extension located in the cytosol. This domain functions as a target site for the protein kinase SOS2, which stimulates SOS1 transport by phosphorylating its autoinhibitory C-terminal tail [18]. This structural peculiarity of SOS1 resembles that of the C-terminal extension found in NHE1, a plasma membrane Na^+^/H^+^ exchanger present in animal cells [26]. Although the C-terminal extension of NHE1 is not nearly as long as the corresponding domain in SOS1, this C-terminal extension binds to a plethora of regulatory proteins, thereby contributing to the fine-tuning of its transport efficiency. This observation prompted us to search for novel SOS1-binding proteins that might be involved in its regulation.

As shown in our pull-down experiments (Table S1), the recombinant C-term protein indeed interacted with many Arabidopsis proteins. Strikingly, among the top 15 candidates, which were ranked according to relative interaction abundance with the bait SOS1 C-term protein, the 14-3-3 protein family was clearly overrepresented, with five members. BiFC experiments successfully revealed protein–protein interactions between SOS1 and the 14-3-3 isoforms ω, κ, υ, and ν (Figure 2A–D), confirming that these interactions are highly likely to occur under cellular conditions in vivo.

We focused on these 14-3-3 proteins not only because they interacted with the SOS1 C-terminal domain in a pull-down experiment and in living cells (Table S1 and Figure 2A–D), but also because 14-3-3 proteins are binding partners of the C-term domain of the human Na^+^/H^+^ Exchanger1 (NHE1, [40]). Human 14-3-3 proteins bind to the prototypical serine recognition site in NHE1, i.e., RSXS_703_XP [40], when the serine residue is phosphorylated. Interestingly, we determined that 14-3-3ω binds to a non-canonical, non-phosphorylated binding site of SOS1, namely the domain ^1112^TRQNTMVESSDEEDEDEG^1129^ (Figure 3A,B). We also tested Ser1138, a known phosphorylation site of SOS1, representing the target amino-acid residue of the protein kinase SOS2 [24]. However, even after we generated a phospho-mimicry mutation at this location, no interaction between 14-3-3ω and the corresponding peptide was detected (Figure 3, lane I 13–14). Although 14-3-3 proteins primarily bind to phosphorylated ligands [34], there is ample evidence that non-phosphorylated domains can also serve as specific binding sites with target proteins [41]. It is worth noting that the 14-3-3ω binding site we identified in SOS1 contains numerous acidic aspartate (D) and glutamate (E) residues (Figure 3B). Thus, these amino acid residues might mimic the negatively charged phosphate groups of phosphoserine-containing ligands in canonical binding domains [42].

Since the recombinant C-term is a target of additional proteins with putative regulatory properties, we reasoned that overexpressing this SOS1 domain might lead to altered salt tolerance in Arabidopsis. Although transforming wild-type plants with the C-term construct led to a number of antibiotic-resistant lines and changes in the corresponding mRNA levels (Figure 4), C-term protein levels were relatively low in these transgenic Arabidopsis plants (Figure S4). However, several independent observations of the transformants indicated that overexpressing the SOS1 C-terminus resulted in increased salt tolerance compared to wild-type plants, including the following changes in response to salt stress: (i) decreased sodium levels in leaves; (ii) a less delayed onset of flowering; (iii) lower levels of compatible solutes such as sugars and proline; and (iv) less starch accumulation in leaves (Figure 5B, Figure 6B, Figure 7C, Figure 8A–D and Figure 8F). We believe that the improved salt tolerance in the p35s::SOS1^c-term^StrepII lines was due to enhanced SOS1 protein activity in these plants. This assumption is based on two independent observations: Firstly, leaves from p35s::^SOS1c^termStrepII plants grown in soil or hydroponic solution in the presence of high salt levels contained less Na^+^ than wild-type plants (Figure 6B and Figure 7C). This finding is in line with observations of Arabidopsis and tobacco mutants with increased SOS1 activity [9,43]. Secondly, *WRKY25*, which is markedly upregulated in response to salt treatment [44], was expressed at substantially lower levels in p35s::SOS1^c-term^StrepII than in wild-type plants upon salt stress (Figure 7E). The observation that *WRKY25* mRNA levels were lower in the roots of p35s::SOS1^c-term^StrepII plants while total sodium levels were comparable to those of wild-type roots (Figure 7D) does not contradict this conclusion, as our approach could not distinguish between apoplastic vs. cytosolic Na^+^ levels. Instead, this observation supports our assumption that a fraction of the overall sodium in p35s::SOS1^c-term^StrepII plants was not present in the cytosol but was instead exported into the apoplast due to increased SOS1 activity [45].

Both the direct quantification of sodium levels in leaves and the analysis of molecular responses demonstrate that the leaves of p35s::SOS1^c-term^StrepII plants contained less Na^+^ than wild-type leaves. There is currently no evidence for the substantial back-transport of salt from leaves to roots via the phloem [1]. Therefore, it is likely that the increased SOS1 activity in the rhizodermis cells of these transformed plants prevented Na^+^ loading into the xylem, ultimately leading to low sodium levels in leaves. This assumption is in line with the observation that roots can re-translocate a significant portion of excess sodium back to the soil, making this process one of four distinct factors determining the degree of sodium loading into the xylem [36,38].

The observation that *p35s::SOS1^c-term^StrepII* plants contained lower levels of sugars and starch than wild-type plants after exposure to salt (Figure 8A–D) indicates that the leaf cells of the transformants did not experience the same level of salt stress as wild-type mesophyll cells, which must ramp up sugar and starch production to withstand changes in osmotic potential [39,46]. Moreover, the sugar and starch levels in *p35s::SOS1^c-term^StrepII* plants are in agreement with the less impaired physiology observed in these plants under salt stress. As a general consequence of impaired physiology, salt-stressed plants show delayed flowering [35,47]. This process leads to the accumulation of carbon (in the form of sugars and starch), which cannot be converted into biomass. However, since salt stress led to less impaired inflorescence formation in *p35s::SOS1^c-term^StrepII* plants (Figure 5A,B), more carbon was converted into biomass in these plants compared to the wild type.

Like sugars, proline levels did not increase to the same extent after salt stress in two of the three *p35s::SOS1^c-term^StrepII* lines compared to the wild type (Figure 8F). In general, various physico-chemical and molecular properties of proline make this amino acid a ubiquitous “stress adaptor molecule” [48]. Accordingly, proline plays an important role in plant salt tolerance. Indeed, the extent of salt-induced deterioration in sos1 loss-of-function mutants is directly correlated with increased proline levels [49]. Moreover, increasing sodium concentrations in all plant tissues are generally accompanied by increased proline levels [50]. Thus, our observation that proline levels were reduced in *p35s::SOS1^c-term^StrepII* lines is in agreement with the reduced sodium levels in the leaves of these plants (Figure 6B and Figure 7C).

C-term SOS1-overexpressing plants grown under control conditions show an inflorescence initiation pattern similar to that of wild-type plants (Figure 5A). However, the salt-induced delay in flowering was less pronounced in the *p35s::SOS1^c-term^StrepII* lines, as revealed at both the morphological and molecular levels (Figure 5B–D; Figure 6B). This difference can be taken as bona fide evidence that overexpressing the C-term of SOS1 confers increased salt tolerance in plants. The moment of inflorescence initiation is delayed with increasing soil salinity in Arabidopsis [35,47]. The delay in inflorescence initiation upon salt stress (Figure 5B) is likely due to at least two processes. Firstly, salt-stressed plants show decreased *FT* and *CONSTANS* expression, and both genes are required for flower induction (Figure 5C,D). Secondly, salt stress results in the degradation of GIGANTEA protein, which leads to SOS1 activation via increased SOS2 kinase activity. In the absence of excess salt, GIGANTEA binds to SOS2, thereby keeping it in an inactivate state [20,51] that prevents uncontrolled SOS1 activation [18]. However, in the presence of excess salt, the binding of GIGANTEA to SOS2 is reduced, allowing the kinase SOS2 to readily activate SOS1 via direct phosphorylation.

We suggest the following mechanism of action for the effects of overexpressing the C-term portion of SOS1: the SOS1 C-term acts as a decoy for binding proteins that normally interact with the native SOS1 carrier to maintain the SOS pathway in an inactive state. Indeed, both 14-3-3 isoforms *kappa* (κ) and *lambda* (λ) inhibit the SOS pathway in Arabidopsis in the absence of salt. The 14-3-3 proteins interact with SOS2 and prevent it from activating SOS1 [21]. Upon salt stress, the 14-3-3/SOS2 complex dissociates and the protein kinase SOS2 is rendered catalytically active [21]. Given that *p35s::SOS1^c-term^StrepII* plants showed increased levels of C-term SOS2 protein (Figure S4), which bound to the four selected 14-3-3 isoforms tested in living cells (Figure 2A–D), we propose that scavenging of the 14-3-3 proteins κ and λ resulted in slightly increased SOS1 activity in the transgenic lines. This increased SOS1 activity is responsible for the improved salt tolerance of SOS1 C-term overexpressing plants.

Given that the *p35s::SOS1^c-term^StrepII* lines exhibit improved salt tolerance (Figure 6B; Figure 7C,E) and that the SOS1 C-term protein is able to bind to various 14-3-3 isoforms in living cells (Figure 2A–D), it is tempting to speculate that 14-3-3 proteins not only interact with and inhibit SOS2 activity, but they also reduce SOS1 transporter activity. In order for this process to be dynamic, a mechanism must exist that triggers the dissociation of 14 3-3 from SOS1 upon the onset of salt stress. In this context, it is worth mentioning that salt stress leads to increased Ca^2+^ levels in plant cells [52], which not only activate SOS3/SOS2 [18], but also increase Ca^2+^-dependent protein kinase (CPK) activity. Indeed, CPK3 phosphorylates 14-3-3 proteins [53], which generally induces a dissociation of functional 14-3-3 dimers and a loss of interaction with target proteins [54,55]. Thus, increased Ca^2+^ levels upon salt stress may not only increase SOS2 activity but also promote the removal of inhibitory 14-3-3 proteins from SOS1 protein. However, neither the exact level of the SOS1 C-term protein nor the exact levels of various 14-3-3 isoforms in Arabidopsis cells are known. Further experiments are needed to determine if SOS1 protein binds sufficient amounts of inhibitory 14-3-3 proteins to inactivate the SOS pathway.

In summary, we have demonstrated that: (a) the SOS1 C-terminal domain physically interacts with previously unknown proteins, most of which belong to the 14-3-3 protein family; (b) overexpressing the SOS1 C-terminal protein domain resulted in increased salt tolerance in transgenic lines, likely via a mechanism that buffers inhibitory binding partners, causing the SOS pathway to assume a more active state. Given that the human NHE1 protein binds to a plethora of important partner proteins, including 14-3-3 proteins [26], it is likely that additional proteins interact with the plant SOS1 C-terminal domain, although their physiological functions remain to be deciphered. The discovery of additional regulatory factors that interfere with the SOS pathway might inspire the development of new biotechnological strategies for improving salt tolerance in agronomically important species, a critical objective in efforts to prevent crop loss in the future.

## 4. Materials and Methods

### 4.1. Plant Material and Growth Conditions

Wild type and mutant Arabidopsis (*Arabidopsis thaliana* (L.) Heynh., ecotype Columbia) plants were grown in a growth chamber at 21 °C (day and night), at 125 µE and short-day conditions (10 h light, 14 h darkness), and alternatively in either liquid culture or hydroponics (see Suppl. Methods).

### 4.2. Generation of p35s::SOS1^c-term^StrepII Plants

To create C-term over-expressor mutants, the SOS1 amino acid sequence 446 to 1146 was cloned as a Strep-tagII-SOS1 C-terminus fusion construct and introduced into the plant genome via the floral dip method (see Suppl. Methods for details).

### 4.3. Gene Expression Analysis

mRNA from Arabidopsis was extracted from frozen leaf and root material using the NucleoSpin^®^ RNA Plant Kit and quantification of selected mRNA species was done as given in the Suppl. Methods, with help of the gene-specific oligonucleotides (Table S3).

### 4.4. Interaction Studies on AtSOS1-C-Terminus Using Bimolecular Fluorescence Complementation (BiFC)

We verified of the interaction between the SOS1-C-terminus and the identified binding partners in vivo via the bimolecular fluorescence complementation (BiFC) method. For this purpose, we generated YFP^N^ and YFP^C^ fusion constructs using the GATEWAY™ specific destination vectors pUBC-cYFP-Dest, pUBC-nYFP-Dest, pUBN-cYFP-Dest, and pUBN-nYFP-Dest (see Suppl. Methods).

### 4.5. Metabolite/Ion Extraction and Quantifications

Sugars, proline, cations, and starch were quantified in plant extracts via chromatography (see Suppl. Methods).

### 4.6. Heterologous Synthesis of AtSOS1 C-Terminus (aa978–1146) and At14-3-3 ω, Protein pURIFICation and Pull-Down Assays

To generate a N-terminal GST-tag fusion construct, the *At*SOS1 C-terminus(aa978–1146) was heterologously expressed in *E.coli* and purified according to standard protocols (see Suppl. Methods). Purification of recombinant proteins and pull-down assays were done according to established methods (see Suppl. Methods).

### 4.7. SDS-PAGE and Immunostaining

SDS-PAGE was performed, and separated proteins were stained with coomassie brilliant blue R 250 or used for immunoblots. Immunodetection was carried out with a monoclonal anti-GST antibody (see Suppl. Methods).

### 4.8. Dot Blot-Analyses

Detection of the recombinant Strep-tagged SOS1 C-terminus protein in *p35s::SOS1^c-term^StrepII* plants, was carried out using salt-stressed Arabidopsis root tissues (see Suppl. Methods).

### 4.9. Peptide-Spot Binding Assay

15 amino acid long peptides, which cover the wild-type sequence of SOS1-C-terminus with an overlap of 12 aa, were synthesized and immobilized on a cellulose membrane, prior to immune-detection using an anti-Histidine antibody (see Suppl. Methods).

## 5. Conclusions

With the demonstration that the C-terminal portion of SOS1 is able to bind regulatory proteins we showed, that this portion of the plant SOS1 carrier protein exhibits similar functions as the corresponding domain of the human homolog NHE. Binding of soluble 14:3:3 proteins to the SOS1 carrier protein maintains the SOS pathway in an inactive state. Further analyses will decipher in more detail how physical interaction of other proteins with the SOS1 C-terminal domain contributes to fine-tuning of the complete SOS pathway, required for salt tolerance in vascular plants.

## Supplementary Materials

Supplementary materials can be found at https://www.mdpi.com/1422-0067/21/9/3334/s1. Figure S1. Eluates from the Affi-Gel10 affinity media columns; Figure S2. Negative control of Bimolecular Fluorescence Complementation analysis in *N. benthamiana*; Figure S3. Expression and purification of the recombinant At14-3-3ω; Figure S4. Protein levels of SOS1 C-terminus; Figure S5. Relative expression levels of SOS1-N-terminus, revealed by quantitative RT-PCR; Table S1. Putative interaction partners of the recombinant AtSOS1-Cterminus protein under control conditions identified by mass-spectrometry; Table S2. Peptide sequences of SOS1 C-terminus (aa446-1146) spotted on Cellulose membrane; Table S3. Oligonucleotides used for the generation of gene constructs and for qRT-PCR.
