# Supplementary material for: 14-3-3 Proteins and Other Candidates form Protein-Protein Interactions with the Cytosolic C-terminal End of SOS1 Affecting Its Transport Activity
Source: Int J Mol Sci. 2020 May 8;21(9):3334. doi: 10.3390/ijms21093334 (PMC7246916; doi:10.3390/ijms21093334)
Supplement: Supplementary file 1 [file ijms-21-03334-s001.pdf]

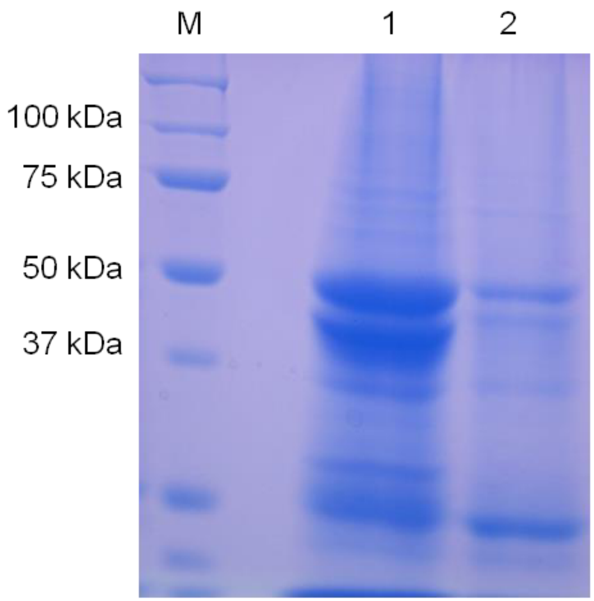

Suppl. Figure S1. Eluates from the Affi-Gel10 affinity media columns. SDS-PAGE of the SDS-Eluates taken from the Affi-Gel10 columns, loaded with either GST alone (2; control) or the SOS1-C-term (aa978-1146) GST-fusion protein (1). Slices of the entire lane were used for mass-spectrometric analyses of bound interaction partners.

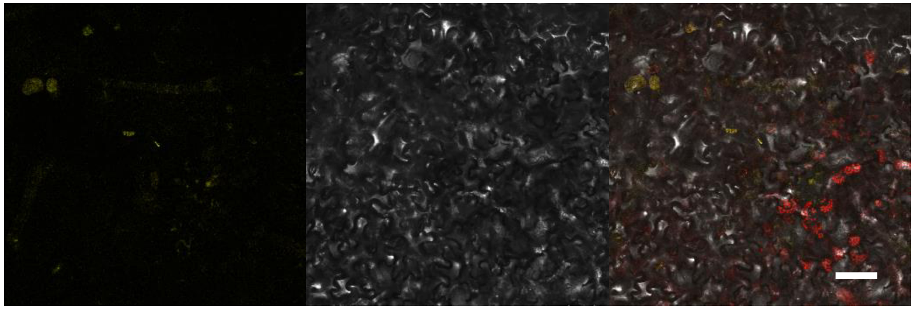

Suppl. Figure S2. Negative control of Bimolecular Fluorescence Complementation analysis in *N. benthamiana*. Plasmids containing the generated *sos1Cterm::yfp*^{NT} and *vik1::yfp*^{CT} constructs were transiently co-transformed into *N. benthamiana* leaves using *A. tumefaciens* mediated transformation. Left image: YFP fluorescence, middle image: transmitted light and right image: merge of YFP fluorescence, chlorophyll fluorescence and transmitted light. The YFP fluorescence signal was detected using a Leica TCS SP5II confocal laser scanning microscope system. Bar = 100 µm.

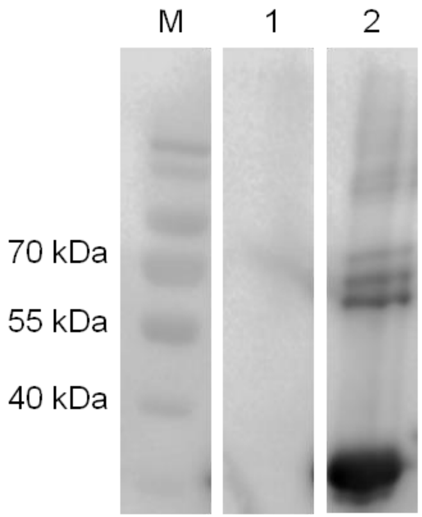

Suppl. Figure S3. Expression and purification of the recombinant *At*14-3-3ω. Western Blot of purified *At*14-3-3ω. Nickel-Sepharose chromatography was performed using a (10x) His-tag fusion protein. 1, total proteins from *At*14-3-3ω expressing *Escherichia coli* cells; 2, purified eluate. The molecular mass (kDa) of the marker proteins is indicated.

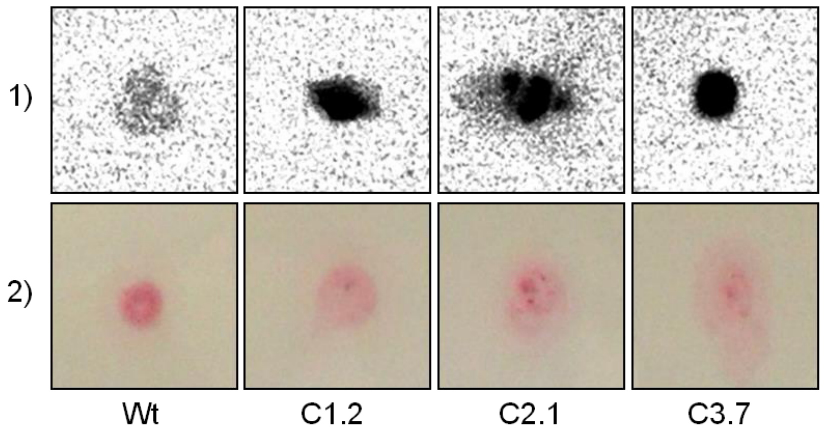

Suppl. Figure S4. Protein levels of *SOS1 C-terminus.* Dot-blot analyses was performed to detect recombinant Strep-tagged SOS1 C-terminus protein in *p35s::SOS1*$^{c\text{-}term}$*StrepII* plants, using salt-stressed Arabidopsis root tissues. After acetone precipitation, supernatants of ground root material were dropped onto a nitrocellulose membrane. Immuno-detection was carried out with a monoclonal anti-Strep·Tag® antibody (1). As a loading control, the blot was stained with Ponceau S solution prior to immunoprinting (2).

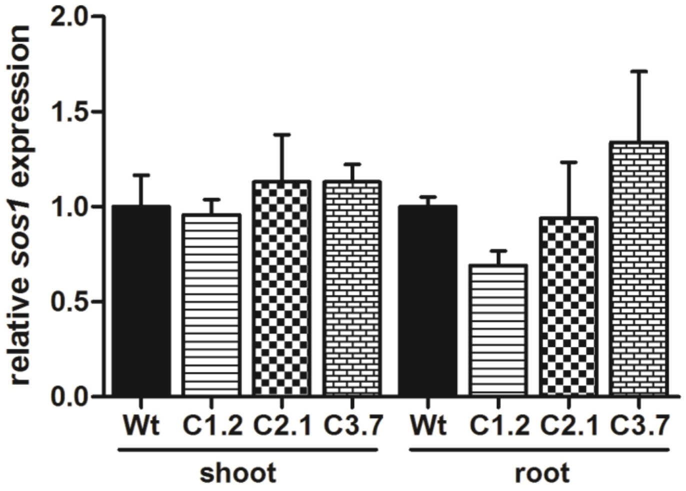

Suppl. Figure S5. Relative expression levels of *SOS1-N-terminus,* revealed by quantitative RT-PCR. Relative transcript levels of endogenous *SOS1 N-terminus* in Arabidopsis root tissues under salt stress conditions. Arabidopsis plants were grown in hydroponic culture for five weeks and watered for three days with 150 mM NaCl. Data represent means, +/- SE, of six independent biological replicates. Data normalized to housekeeping gene *pp2a* (*At1g13320*).

Table S1. Putative interaction partners of the recombinant *At*SOS1-Cterminus protein under control conditions identified by mass-spectrometry.

Gene number	Predicted protein function
AT3G23810	SAHH2 (Adenosylhomocysteinase 2); Protein is involved in a subpathway that synthesizes L-homocysteine from S-adenosyl-L-homocysteine, decreased activity might increase drought tolerance (Li et al. 2015, Front. Plant Sci 6: 717)
AT4G20890	TUBB9 (Tubulin beta-9 chain); Structural constituent of cytoskeleton, functions in microtubule-based process
AT5G62690	TUBB 2 (Tubulin beta-2 chain); Structural constituent of cytoskeleton, functions in microtubule-based process
AT5G19770	TUBA3 (Tubulin alpha-3 chain); Structural constituent of cytoskeleton, functions in microtubule-based process
AT1G20010	TUBB5 (Tubulin beta-5 chain); Structural constituent of cytoskeleton, functions in microtubule-based process
AT1G78300.1	**14-3-3-like protein G-Box Factor 14 omega**, General Regulatory Factor 2, involved in brassinosteroid mediated signaling pathway, located in Golgi apparatus, cytoplasm, cytosol, plasma membrane, vacuolar membrane, vacuole, functions in protein phosphorylated amino acid binding
AT1G35160.2	14-3-3-like protein GF14 phi, General Regulatory Factor 4, involved in brassinosteroid mediated signaling pathway, located in Golgi apparatur, cytoplasm, cytosol, nuclear envelope, nucleus, plasma membrane, functions in protein phosphorylated amino acid binding
AT2G36530	ENO2 (Bifunctional enolase 2/transcriptional activator); Multifunctional enzyme that acts as an enolase involved in the metabolism and as a positive regulator of cold-responsive gene transcription, involved in a subpathway that synthesizes pyruvate from D-glyceraldehyde 3-phosphate. This subpathway is part of the pathway glycolysis, which is itself part of Carbohydrate degradation
AT3G55610	Delta-1-pyrroline-5-carboxylate synthase P5CS plays a key role in proline biosynthesis, leading to osmoregulation in plants (Hmida-Sayari et al. 2005 Plant Sci. 169: 746-752)
AT5G65430.3	**14-3-3-like protein GF14 kappa**, General Regulatory Factor 8, involved in brassinosteroid mediated signalling pathway, located in chloroplast, cytoplasm, nucleus, plant-type cell wall, plasma membrane, functions in protein phosphorylated amino acid binding
AT1G02500	SAM1 (S-adenosylmethionine synthase 1); Catalyzes the formation of S-adenosyl-methionine from methionine and ATP. This protein is involved in a subpathway that synthesizes S-adenosyl-L-methionine from L-methionine. This subpathway is part of the pathway S-adenosyl-L-methionine biosynthesis, which is itself part of Amino-acid biosynthesis
AT5G16050	**14-3-3-like protein GF14 upsilon**, General Regulatory Factor 5, involved in the response to cadmium ion, located in Golgi apparatus, cell wall, chloroplast, stroma, cytoplasm, cytosol, mitochondrion, nuclear envelope, plasma membrane, involved in brassinosteroid mediated signaling pathway, functions in protein phosphorylated amino acid binding
AT3G02520.1	14-3-3-like protein GF14 nu, General Regulatory Factor 7, involved in brassinosteroid mediated signaling pathway, functions in protein phosphorylated amino acid binding
ATMG01190	ATPA (ATP synthase subunit alpha, mitochondrial); Functions in ATP hydrolysis coupled proton transport

AT2G16950	TRN1 (Transportin-1); Functions in intracellular protein transport, nuclear protein import as nuclear transport receptor
**Gene number**	**Predicted protein function**
AT2G36880	METK3 (S-adenosylmethionine synthase 3); Protein is involved in a subpathway that synthesizes S-adenosyl-L-methionine from L-methionine. This subpathway is part of the pathway S-adenosyl-L-methionine biosynthesis, which is itself part of Amino-acid biosynthesis
AT5G35360	CAC2 (Biotin carboxylase, chloroplastic); Protein is involved in a subpathway that synthesizes malonyl-CoA from acetyl-CoA
AT1G12840	VHA-C (V-type proton ATPase subunit C); Subunit of the peripheral V1 complex of vacuolar ATPase. Subunit C is necessary for the assembly of the catalytic sector of the enzyme and is likely to have a specific function in its catalytic activity. V-ATPase is responsible for acidifying a variety of intracellular compartments in eukaryotic cells. ATP hydrolysis coupled proton transport
AT1G76550	PFP-alpha2 (Pyrophosphate--fructose 6-phosphate 1-phosphotransferase subunit alpha 2); Protein is involved in a subpathway that synthesizes D-glyceraldehyde 3-phosphate and glycerone phosphate from D-glucose This subpathway is part of the pathway glycolysis, which is itself part of Carbohydrate degradation
AT1G22300.1	14-3-3-like protein GF14 epsilon, General Regulatory Factor 10, involved in the response to ABA, located in chloroplast, stroma, cytoplasm, cytosol, mitochondrion, plasma membrane, plasmodesma, involved in brassinosteroid mediated signaling pathway, functions in protein phosphorylated amino acid binding
AT2G05710	ACO2 (Aconitate hydratase 2, mitochondrial); Protein is involved in a subpathway that synthesizes isocitrate from oxaloacetate. This subpathway is part of the pathway tricarboxylic acid cycle, which is itself part of Carbohydrate metabolism
AT5G15450	CLPB3 (Chaperone protein, chloroplastic); Molecular chaperone essential for chloroplast development and seedling viability
AT1G53240	MDH1 (Malate dehydrogenase 1, mitochondrial)
AT1G09795	ATP phosphoribosyltransferase 2, chloroplastic; Protein is involved in a subpathway that synthesizes L-histidine from 5-phospho-alpha-D-ribose 1-diphosphate. This subpathway is part of the pathway L-histidine biosynthesis, which is itself part of Amino-acid biosynthesis
AT5G08280	Porphobilinogen deaminase, chloroplastic; Protein is involved in a subpathway that synthesizes coproporphyrinogen-III from 5-aminolevulinate. This subpathway is part of the pathway protoporphyrin-IX biosynthesis, which is itself part of Porphyrin-containing compound metabolism
AT1G18500	IPMS1 (2-isopropylmalate synthase 1, chloroplastic); Protein is involved in a subpathway that synthesizes L-leucine from 3-methyl-2-oxobutanoate. This subpathway is part of the pathway L-leucine biosynthesis, which is itself part of Amino-acid biosynthesis
AT2G42590.3	14-3-3-like protein GF14 mu, General Regulatory Factor 9, involved in post-embryonic root development, located in chloroplast, stroma, cytoplasm, cytosol, nucleus, plasma membrane, functions in calcium ion binding, protein phosphorylated amino acid binding
AT2G25450	GSL-OH (Probable 2-oxoacid dependent dioxygenase); 1-aminocyclopropane-1-carboxylate oxidase activity
AT2G45300	3-phosphoshikimate 1-carboxyvinyltransferase, chloroplastic; Protein is involved in a subpathway that synthesizes chorismate from D-erythrose 4-phosphate and phosphoenolpyruvate.
AT2G20580	RPN2A (26S proteasome non-ATPase regulatory subunit 2 homolog A); functions in ubiquitin binding

AT1G32060	Phosphoribulokinase, chloroplastic; Protein is involved in the pathway Calvin cycle, which is part of Carbohydrate biosynthesis
**Gene number**	**Predicted protein function**
AT3G20050	CCT1 (T-complex protein 1 subunit alpha); molecular chaperone, functions in ATP binding
AT4G21210	RP1 (Pyruvate, phosphate dikinase regulatory protein, chloroplastic); Bifunctional serine/threonine kinase and phosphorylase involved in the dark/light-mediated regulation of PPDK by catalyzing its phosphorylation/dephosphorylation
AT1G05010	ACO4 (1-aminocyclopropane-1-carboxylate oxidase 4); Protein is involved in a subpathway that synthesizes ethylene from S-adenosyl-L-methionine. This subpathway is part of the pathway ethylene biosynthesis via S-adenosyl-L-methionine, which is itself part of Alkene biosynthesis
AT5G55280	FtsZ1 (Cell division protein homolog 1, chloroplastic); Exhibits GTPase activity
AT5G20630	GER3 (Germin-like protein subfamily 3 member 3)
AT3G14790	RHM3 (Probable rhamnose biosynthetic enzyme 3); Involved in Carbohydrate biosynthesis
AT5G17990	PAT1 (Anthranilate phosphoribosyltransferase, chloroplastic); Protein is involved in a subpathway that synthesizes L-tryptophan from chorismate
AT5G49460	ACLB-2 (ATP-citrate synthase beta chain protein 2); Primary enzyme responsible for the synthesis of cytosolic acetyl-CoA, used for the elongation of fatty acids and biosynthesis of isoprenoids, flavonoids and malonated derivatives
AT2G40490	UPD2 (Uroporphyrinogen decarboxylase 2, chloroplastic); Protein is involved in a subpathway that synthesizes coproporphyrinogen-III from 5-aminolevulinate
AT1G06680	OEE2 (Oxygen-evolving enhancer protein 2-1, chloroplastic); May be involved in the regulation of photosystem II
AT1G17050	SPS2 (Solanesyl diphosphate synthase 2, chloroplastic); Involved in isoprenoid biosynthetic pathway
AT2G30110	UBA1 (Ubiquitin-activating enzyme E1-1); Involved in the pathway protein ubiquitination, which is part of Protein modification
AT4G23100	GSH1 (Glutamate--cysteine ligase, chloroplastic); protein is involved in a subpathway that synthesizes glutathione from L-cysteine and L-glutamate
AT1G57720	Probable elongation factor 1-gamma 2, translation elongation factor activity
AT1G64520	RPN12A (26S proteasome non-ATPase regulatory subunit 8 homolog A); Acts as a regulatory subunit of the 26S proteasome which is involved in the ATP-dependent degradation of ubiquitinated protein
AT4G23460	Beta-adaptin-like protein C; Subunit of chlathrin-associated adaptor protein complex
AT4G29120	Probable 3-hydroxyisobutyrate dehydrogenase-like 1, mitochondrial; Protein is involved in the pathway L-valine degradation, which is part of Amino-acid degradation
AT1G76080	CDSP32 (Thioredoxin-like protein, chloroplastic); Probable thiol-disulfide oxidoreductase involved in resistance to oxidative stress
AT5G01410	PDX1.3 (Pyridoxal biosynthesis protein); Protein is involved in the pathway pyridoxal 5'-phosphate biosynthesis, which is part of Cofactor biosynthesis
AT5G58290	RPT3 (26S protease regulatory subunit 6B homolog); Protein is involved in the ATP-dependent degradation of ubiquitinated protein
AT3G14390	LYSA1 (Diaminopimelate decarboxylase 1, chloroplastic); Protein is involved in a subpathway that synthesizes L-lysine from DL-2,6-diaminopimelate. This

	subpathway is part of the pathway L-lysine biosynthesis via DAP pathway, which is itself part of Amino-acid biosynthesis
**Gene number**	**Predicted protein function**
AT1G03630	PORC (Protochlorophyllide reductase C, chloroplastic); Protein is involved in the pathway chlorophyll biosynthesis, which is part of Porphyrin-containing compound metabolism
AT4G24450	GWD2 (Alpha-glucan water dikinase 2); Mediates the incorporation of phosphate into alpha-glucan
AT1G12410	CLPR2 (ATP-dependent protease proteolytic subunit-related protein 2, chloroplastic); Required for chloroplast development and integrity
AT5G22800	EMB86 (Probable alanine--tRNA ligase, chloroplastic); Catalyzes the attachment of alanine to tRNA(Ala)
AT5G03940	FFC (Signal recognition particle 54 kDa protein, chloroplastic); Involved in cotranslational and post-translational sorting of thylakoid proteins
AT1G01470	LEA14 (late embryogenesis abundant protein 14); Probable desiccation-related protein
AT3G10920	MSD1 (Superoxide dismutase [Mn] 1, mitochondrial); Destroys superoxide anion radicals
AT5G45930	CHLI2 (Magnesium-chelatase subunit ChlI-2, chloroplastic); Protein is involved in the pathway chlorophyll biosynthesis, which is part of Porphyrin-containing compound metabolism.
AT5G37830	OXP1 (5-oxoprolinase); Acts in glutathione degradation pathway
AT3G19170	PreP1 (Presequence protease 1, chloroplastic/mitochondrial); ATP-independent protease that degrades both mitrochondrial and chloroplastic transit peptides after their cleavage
AT5G62790	DXR (1-deoxy-D-xylulose 5-phosphate reductoisomerase, chloroplastic); Protein is involved in step **1** of the subpathway that synthesizes isopentenyl diphosphate from 1-deoxy-D-xylulose 5-phosphate
AT4G33510	DHS2 (Phospho-2-dehydro-3-deoxyheptonate aldolase 2, chloroplastic); Protein is involved in step **1** of the subpathway that synthesizes chorismate from D-erythrose 4-phosphate and phosphoenolpyruvate.
AT4G31490	Coatomer subunit beta-2; Coatomer complex is required for budding from Golgi membranes
AT1G79870	HPR2 (Glyoxylate/hydroxypyruvate reductase A); Catalyzes the NADPH-dependent reduction of glyoxylate and hydroxypyruvate into glycolate and glycerate
AT3G59970	MTHFR1 (Methylenetetrahydrofolate reductase 1); Protein is involved in the pathway tetrahydrofolate interconversion, which is part of One-carbon metabolism
AT3G25800	PP2AA2 (Serine/threonine-protein phosphatase 2A, 65 kDa regulatory subunit A beta isoform); Subunit serves a scaffolding molecule to coordinate the assembly of the catalytic domain and a variable regulatory domain
AT2G24270	ALDH11A3 (NADP-dependent glyceraldehyde-3-phosphate dehydrogenase); Important as a means of generating NADPH for biosynthetic processes
AT1G47128	RD21a (Cysteine proteinase); Cysteine protease that plays a role in immunity, senescence, biotic and abiotic stresses
AT2G46520	CAS (Exportin-2); Mediates importin-alpha re-export from the nucleus to the cytoplasm
AT1G80460	GLPK (Glycerol kinase); Key enzyme in the regulation of glycerol uptake and metabolism

AT5G26570	GWD3 (Phosphoglucan, water dikinase, chloroplastic); Mediates the incorporation of phosphate into phospho-alpha-glucan, mostly at the C3 position of the glucose unit, required for starch degradation
**Gene number**	**Predicted protein function**
AT5G28840	GDP-mannose 3,5-epimerase; protein is involved in a subpathway that synthesizes L-ascorbate from GDP-alpha-D-mannose
AT2G25080	GPX1 (Phospholipid hydroperoxide glutathione peroxidase 1, chloroplastic); Protects cells and enzymes from oxidative damage by catalyzing the reduction of hydrogen peroxide
AT3G22890	APS1 (ATP sulfurylase 1, chloroplastic); Protein is involved in a subpathway that synthesizes sulfite from sulfate
AT4G26900	HISN4 (Imidazole glycerol phosphate synthase hisHF, chloroplastic); Protein is involved in a subpathway that synthesizes L-histidine from 5-phospho-alpha-D-ribose 1-diphosphate. This subpathway is part of the pathway L-histidine biosynthesis, which is itself part of Amino-acid biosynthesis
AT5G52920	PKP2 (Plastidial pyruvate kinase 2); Required for plastidial pyruvate kinase activity
AT3G14990	DJ1A (Protein DJ-1 homolog A); Involved in oxidate stress response
AT2G27530	RPL10AB (60S ribosomal protein L10a-2)
AT1G02930	GSTF6 (Glutathione S-transferase F6); May be involved in the conjugation of reduced glutathione to endogenous and exogenous hydrophobic electrophiles
AT5G54160	OMT1 (Flavone 3'-O-methyltransferase 1); Protein is involved in the pathway quercetin degradation, which is part of Flavonoid metabolism
AT2G35500	SKL2 (Probable inactive shikimate kinase like 2, chloroplastic)
AT5G40390	RFS5 (Probable galactinol-sucrose galactosyltransferase 5); Transglucosidase operating by a ping-pong reaction mechanism
AT2G29450	GSTU5 (Glutathione S-transferase U5); May be involved in the conjugation of reduced glutathione to endogenous and exogenous hydrophobic electrophiles
AT1G07320	RPL4 (50S ribosomal protein L4, chloroplastic); Protein binds directly and specifically to 23S rRNA
ATCG00380	rps4 (30S ribosomal protein S4, chloroplastic); One of the primary rRNA binding proteins
AT5G36160	TAT (Tyrosine aminotransferase); Protein is involved in step **2** of the subpathway that synthesizes acetoacetate and fumarate from L-phenylalanine. This subpathway is part of the pathway L-phenylalanine degradation, which is itself part of Amino-acid degradation
AT5G66570	OEE1 (Oxygen-evolving enhancer protein 1-1, chloroplastic); Stabilizes the manganese cluster which is the primary site of water splitting
AT1G14030	LSMT-L ([Fructose-bisphosphate aldolase]-lysine N-methyltransferase, chloroplastic); Protein-lysine methyltransferase methylating chloroplastic fructose 1,6-bisphosphate aldolases
AT4G04040	PFP-beta2 (Pyrophosphate--fructose 6-phosphate 1-phosphotransferase subunit beta 2); Catalyzes the phosphorylation of D-fructose 6-phosphate
ATCG00830<br>ATCG01310	rpl2-A (50S ribosomal protein L2, chloroplastic); Structural constituent of ribosome
AT1G58080	ATP-PRTase 1 (ATP phosphoribosyltransferase 1, chloroplastic); Protein is involved in a subpathway that synthesizes L-histidine from 5-phospho-alpha-D-ribose 1-diphosphate

AT4G04020	PAP1 (Probable plastid-lipid-associated protein 1, chloroplastic); Probably involved in light/cold stress-related jasmonate biosynthesis
AT4G19710	AKHSDH2 (Bifunctional aspartokinase/homoserine dehydrogenase 2, chloroplastic)
**Gene number**	**Predicted protein function**
AT5G42740	PGIC (Glucose-6-phosphate isomerase, cytosolic); Protein is involved in a subpathway that synthesizes D-glyceraldehyde 3-phosphate and glycerone phosphate from D-glucose
AT5G53460	GLT1 (Glutamate synthase 1 [NADH], chloroplastic); Involved in glutamate biosynthesis
AT3G58140	Phenylalanine--tRNA ligase, chloroplastic/mitochondrial; Is responsible for the charging of tRNA(Phe) with Phenylalanine in mitochondrial translation
AT1G17650	GLYR2 (Glyoxylate/succinic semialdehyde reductase 2, chloroplastic); Catalyzes the NADPH-dependent reduction of glyoxylate to glycolate
AT4G34490	CAP1 (Cyclase-associated protein 1); Actin monomer binding protein
AT1G68590	30S ribosomal protein 3-1, chloroplastic; probably a ribosomal protein
AT1G24020	MLP423 (MLP-like protein 423); Involved in response to biotic stimulus
AT2G43090	3-isopropylmalate dehydratase small subunit 3; Plays an essential role in leucine biosynthesis
AT2G19940	Probable N-acetyl-gamma-glutamyl-phosphate reductase, chloroplastic
AT3G58500	PP2A3 (Serine/threonine-protein phosphatase PP2A-3 catalytic subunit); Phosphoprotein phosphatase activity
AT2G20890	THF1 (Protein THYLAKOID FORMATION 1, chloroplastic); Involved in the process of vesicle-mediated thylakoid membrane biogenesis
AT1G47340	F-box protein
AT3G19980	FYPP3 (Phytochrome-associated serine/threonine-protein phosphatase 3); Dephosphorylates phosphorylated phytochromes
AT5G48300	APS1 (Glucose-1-phosphate adenylyltransferase small subunit, chloroplastic); Protein plays a role in synthesis of starch
AT5G65620	OOP (Organellar oligopeptidase A, chloroplastic/mitochondrial); Protein plays a role in the degradation of transit peptides
AT3G04880	DRT102 (DNA-damage-repair/toleration protein); Involved in response to cold
AT2G43910	HOL1 (Thiocyanate methyltransferase 1); Involved in glucosinolate metabolism
AT5G27470	Serine--tRNA ligase; Catalyzes the attachment of serine to tRNA(Ser)
AT3G29360	UGD2 (UDP-glucose 6-dehydrogenase 2); Protein is involved in a subpathway that synthesizes UDP-alpha-D-glucuronate from UDP-alpha-D-glucose
AT3G54900	GRXS14 (Monothiol glutaredoxin-S14, chloroplastic); Probably involved in the regulation of the redox state of proteins
AT2G17790	VPS35A (Vacuolar protein sorting-associated protein 35A); Protein plays a role in vesicular protein sorting
AT3G10670	ABCI6 (ABC transporter I family member 6, chloroplastic); ATPase activity coupled to transmembrane transport of substances

AT4G24820	RPN7 (26S proteasome non-ATPase regulatory subunit 6 homolog); Acts as a regulatory subunit of the 26S proteasome which is involved in the ATP-dependent degradation of ubiquitinated proteins
AT5G05780	RPN8A (26S proteasome non-ATPase regulatory subunit 7 homolog A); Acts as a regulatory subunit of the 26S proteasome which is involved in the ATP-dependent degradation of ubiquitinated proteins
**Gene number**	**Predicted protein function**
AT3G54640	TSA1 (Tryptophan synthase alpha chain, chloroplastic); Protein is involved in step **5** of the subpathway that synthesizes L-tryptophan from chorismate
AT3G13330	PA200 (Proteasome activator subunit 4); Component of the proteasome
AT2G47390	GEP (Probable glutamyl endopeptidase, chloroplastic); Serine-type protease
AT1G36160	ACC1 (Acetyl-CoA carboxylase 1); Protein is involved in a subpathway that synthesizes malonyl-CoA from acetyl-CoA. This subpathway is part of the pathway malonyl-CoA biosynthesis, which is itself part of Lipid metabolism
AT1G16460	RDH2 (Thiosulfate/3-mercaptopyruvate sulfurtransferase 2); Catalyzes the transfer from a sulfur ion to cyanide or another thiol compound
AT5G59880	ADF3 (Actin-depolymerizing factor 3); Actin-depolymerizing protein
AT5G47200	RABD2b (Ras-related protein); Regulator of membrane traffic from the Golgi apparatur towards the ER
AT3G53580	DAPF (Diaminopimelate epimerase, chloroplastic); Protein is involved in a subpathway that synthesizes DL-2,6-diaminopimelate from LL-2,6-diaminopimelate
AT4G13430	IIL1 (3-isopropylmalate dehydratase large subunit); Protein is involved in a subpathway that synthesizes L-leucine from 3-methyl-2-oxobutanoate
AT3G02360	6-phosphogluconate dehydrogenase, decarboxylating 3; Protein is involved in a subpathway that synthesizes D-ribulose 5-phosphate from D-glucose 6-phosphate
AT3G28300	UPF0496 protein; Integral component of membrane
AT3G48990	4CL8 (4-coumarate--CoA ligase-isomerase 8); Required for oxalate degradation
AT4G12060	Clp protease-related protein, chloroplastic; Protein histidine kinase binding
AT5G64050	GluRS (Glutamate--tRNA ligase, chloroplastic/mitochondrial); Catalyzes the attachment of glutamate to tRNA(Glu)
AT1G80380	GLYK (D-glycerate 3-kinase, chloroplastic); Protein is involved in a subpathway that synthesizes 3-phospho-D-glycerate from glycine
AT5G22580	Stress-response A/B barrel domain-containing protein; Involved in stress response
AT3G43300	BIG5 (Brefeldin A-inhibited guanine nucleotide-exchange protein 5); Plays a role in vesicular protein sorting
AT1G72550	Probable phenylalanine-tRNA ligase beta subunit; Phenylalanine tRNA ligase activity
AT3G63170	FAP1 (Fatty-acid-binding protein 1)
AT1G30530	UGT78D1 (UDP-glycosyltransferase 78D1); UDP-glycosyltransferase activity
AT1G49970	CLPR1 (ATP-dependent Clp protease proteolytic subunit-related protein 1, chloroplastic); Required for chloroplast development and differentiation
AT4G32520	SHM3 (Serine hydroxymethyltransferase 3, chloroplastic); Protein is involved in the pathway tetrahydrofolate interconversion, which is part of One-carbon metabolism
AT1G43670	FBPase (Fructose-1,6-bisphosphatase, cytosolic)

Table S2. Peptide sequences of SOS1 C-terminus (aa446-1146) spotted on Cellulose membrane.

Spot Number	SOS1 C-term Sequence
A1	FVLRLLRMDILPAPK
A2	RLLRMDILPAPKKRI
A3	RMDILPAPKKRILEY
A4	ILPAPKKRILEYTKY
A5	APKKRILEYTKYEML
A6	KRILEYTKYEMLNKA
A7	LEYTKYEMLNKALRA
A8	TKYEMLNKALRAFQD
A9	EMLNKALRAFQDLGD
A10	NKALRAFQDLGDDEE
A11	LRAFQDLGDDEELGP
A12	FQDLGDDEELGPADW
A13	LGDDEELGPADWPTV
A14	DEELGPADWPTVESY
A15	LGPADWPTVESYISS
A16	ADWPTVESYISSLKG
A17	PTVESYISSLKGSEG
A18	ESYISSLKGSEGELV
A19	ISSLKGSEGELVHHP
A20	LKGSEGELVHHPHNG
A21	SEGELVHHPHNGSKI
A22	ELVHHPHNGSKIGSL
A23	HHPHNGSKIGSLDPK
A24	HNGSKIGSLDPKSLK
A25	SKIGSLDPKSLKDIR
A26	GSLDPKSLKDIRMRF
A27	DPKSLKDIRMRFLNG
A28	SLKDIRMRFLNGVQA
A29	DIRMRFLNGVQATYW
A30	MRFLNGVQATYWEML
B1	LNGVQATYWEMLDEG
B2	VQATYWEMLDEGRIS
B3	TYWEMLDEGRISEVT
B4	EMLDEGRISEVTANI
B5	DEGRISEVTANILMQ
B6	RISEVTANILMQSVD
B7	EVTANILMQSVDEAL
B8	ANILMQSVDEALDQV
B9	LMQSVDEALDQVSTT
B10	SVDEALDQVSTTLCD
B11	EALDQVSTTLCDWRG
B12	DQVSTTLCDWRGLKP
B13	STTLCDWRGLKPHVN
B14	LCDWRGLKPHVNFPN
B15	WRGLKPHVNFPNYYN
B16	LKPHVNFPNYYNFLH
B17	HVNFPNYYNFLHSKV

B18	FPNYYNFLHSKVVPR
B19	YYNFLHSKVVPRKLV
B20	FLHSKVVPRKLVTYF
B21	SKVVPRKLVTYFAVE
B22	VPRKLVTYFAVERLE
B23	KLVTYFAVERLESAC
B24	TYFAVERLESACYIS
B25	AVERLESACYISAAF
B26	RLESACYISAAFLRA
B27	SACYISAAFLRAHTI
B28	YISAAFLRAHTIARQ
B29	AAFLRAHTIARQQLY
B30	LRAHTIARQQLYDFL
C1	HTIARQQLYDFLGES
C2	ARQQLYDFLGESNIG
C3	QLYDFLGESNIGSIV
C4	DFLGESNIGSIVINE
C5	GESNIGSIVINESEK
C6	NIGSIVINESEKEGE
C7	SIVINESEKEGEEAK
C8	INESEKEGEEAKKFL
C9	SEKEGEEAKKFLEKV
C10	EGEEAKKFLEKVRSS
C11	EAKKFLEKVRSSFPQ
C12	KFLEKVRSSFPQVLR
C13	EKVRSSFPQVLRVVK
C14	RSSFPQVLRVVKTKQ
C15	FPQVLRVVKTKQVTY
C16	VLRVVKTKQVTYSVL
C17	VVKTKQVTYSVLNHL

C18	TKQVTYSVLNHLLGY
C19	VTYSVLNHLLGYIEN
C20	SVLNHLLGYIENLEK
C21	NHLLGYIENLEKVGL
C22	LGYIENLEKVGLLEE
C23	IENLEKVGLLEEKEI
C24	LEKVGLLEEKEIAHL
C25	VGLLEEKEIAHLHDA
C26	LEEKEIAHLHDAVQT
C27	KEIAHLHDAVQTGLK
C28	AHLHDAVQTGLKKLL
C29	HDAVQTGLKKLLRNP
C30	VQTGLKKLLRNPPIV
D1	GLKKLLRNPPIVKLP
D2	KLLRNPPIVKLPKLS
D3	RNPPIVKLPKLSDMI
D4	PIVKLPKLSDMITSH
D5	KLPKLSDMITSHPLS
D6	KLSDMITSHPLSVAL
D7	DMITSHPLSVALPPA
D8	TSHPLSVALPPAFCE
D9	PLSVALPPAFCEPLK
D10	VALPPAFCEPLKHSK
D11	PPAFCEPLKHSKKEP
D12	FCEPLKHSKKEPMKL
D13	PLKHSKKEPMKLRGV
D14	HSKKEPMKLRGVTLY
D15	KEPMKLRGVTLYKEG
D16	MKLRGVTLYKEGSKP
D17	RGVTLYKEGSKPTGV

D18	TLYKEGSKPTGVWLI
D19	KEGSKPTGVWLIFDG
D20	SKPTGVWLIFDGIVK
D21	TGVWLIFDGIVKWKS
D22	WLIFDGIVKWKSKIL
D23	FDGIVKWKSKILSNN
D24	IVKWKSKILSNNHSL
D25	WKSKILSNNHSLHPT
D26	KILSNNHSLHPTFSH
D27	SNNHSLHPTFSHGST
D28	HSLHPTFSHGSTLGL
D29	HPTFSHGSTLGLYEV
D30	FSHGSTLGLYEVLTG
E1	GSTLGLYEVLTGKPY
E2	LGLYEVLTGKPYLCD
E3	YEVLTGKPYLCDLIT
E4	LTGKPYLCDLITDSM
E5	KPYLCDLITDSMVLC
E6	LCDLITDSMVLCFFI
E7	LITDSMVLCFFIDSE
E8	DSMVLCFFIDSEKIL
E9	VLCFFIDSEKILSLQ
E10	FFIDSEKILSLQSDS
E11	DSEKILSLQSDSTID
E12	KILSLQSDSTIDDFL
E13	SLQSDSTIDDFLWQE
E14	SDSTIDDFLWQESAL
E15	TIDDFLWQESALVLL
E16	DFLWQESALVLLKLL
E17	WQESALVLLKLLRPQ

E18	SALVLLKLLRPQIFE
E19	VLLKLLRPQIFESVA
E20	KLLRPQIFESVAMQE
E21	RPQIFESVAMQELRA
E22	IFESVAMQELRALVS
E23	SVAMQELRALVSTES
E24	MQELRALVSTESSKL
E25	LRALVSTESSKLTTY
E26	LVSTESSKLTTYVTG
E27	TESSKLTTYVTGESI
E28	SKLTTYVTGESIEID
E29	TTYVTGESIEIDCNS
E30	VTGESIEIDCNSIGL
F1	ESIEIDCNSIGLLLE
F2	EIDCNSIGLLLEGFV
F3	CNSIGLLLEGFVKPV
F4	IGLLLEGFVKPVGIK
F5	LLEGFVKPVGIKEEL
F6	GFVKPVGIKEELISS
F7	KPVGIKEELISSPAA
F8	GIKEELISSPAALSP
F9	EELISSPAALSPSNG
F10	ISSPAALSPSNGNQS
F11	PAALSPSNGNQSFHN
F12	LSPSNGNQSFHNSSE
F13	SNGNQSFHNSSEASG
F14	NQSFHNSSEASGIMR
F15	FHNSSEASGIMRVSF
F16	SSEASGIMRVSFSQQ
F17	ASGIMRVSFSQQATQ

F18	IMRVSFSQQATQYIV
F19	VSFSQQATQYIVETR
F20	SQQATQYIVETRARA
F21	ATQYIVETRARAIIF
F22	YIVETRARAIIFNIG
F23	ETRARAIIFNIGAFG
F24	ARAIIFNIGAFGADR
F25	IIFNIGAFGADRTLH
F26	NIGAFGADRTLHRRP
F27	AFGADRTLHRRPSSL
F28	ADRTLHRRPSSLTPP
F29	TLHRRPSSLTPPRSS
F30	RRPSSLTPPRSSSSD
G1	SSLTPPRSSSSDQLQ
G2	TPPRSSSSDQLQRSF
G3	RSSSSDQLQRSFRKE
G4	SSDQLQRSFRKEHRG
G5	QLQRSFRKEHRGLMS
G6	RSFRKEHRGLMSWPE
G7	RKEHRGLMSWPENIY
G8	HRGLMSWPENIYAKQ
G9	LMSWPENIYAKQQQE
G10	WPENIYAKQQQEINK
G11	NIYAKQQQEINKTTL
G12	AKQQQEINKTTLSLS
G13	QQEINKTTLSLSERA
G14	INKTTLSLSERAMQL
G15	TTLSLSERAMQLSIF
G16	SLSERAMYQSIFGSM
G17	ERAMYQSIFGSMVNV

G18	MYQSIFGSMVNVYRR
G19	SIFGSMVNVYRRSVS
G20	GSMVNVYRRSVSFGG
G21	VNVYRRSVSFGGIYN
G22	YRRSVSFGGIYNNKL
G23	SVSFGGIYNNKLQDN
G24	FGGIYNNKLQDNLLY
G25	IYNNKLQDNLLYKKL
G26	NKLQDNLLYKKLPLN
G27	QDNLLYKKLPLNPAQ
G28	LLYKKLPLNPAQGLV
G29	KKLPLNPAQGLVSAK
G30	PLNPAQGLVSAKSES
H1	PAQGLVSAKSESSIV
H2	GLVSAKSESSIVTKK
H3	SAKSESSIVTKKQLE
H4	SESSIVTKKQLETRK
H5	SIVTKKQLETRKHAC
H6	TKKQLETRKHACQLP
H7	QLETRKHACQLPLKG
H8	TRKHACQLPLKGESS
H9	HACQLPLKGESSTRQ
H10	QLPLKGESSTRQNTM
H11	LKGESSTRQNTMVES
H12	ESSTRQNTMVESSDE
H13	TRQNTMVESSDEEDE
H14	NTMVESSDEEDEDEG
H15	VESSDEEDEDEGIVV
H16	SDEEDEDEGIVVRID
H17	EDEDEGIVVRIDSPS

H18	DEGIVVRIDSPSKIV
H19	IVVRIDSPSKIVFRN
H20	VRIDSPSKIVFRNDL
H21	-
H22	-
H23	-
H24	-
H25	-
H26	-
H27	-
H28	-
H29	-
H30	-
I1	-
I2	-
I3	-
I4	-
I5	IVTKKQLETRKHACQ
I6	KKQLETRKHACQLPL
I7	LETRKHACQLPLKGE
I8	RKHACQLPLKGESST
I9	ACQLPLKGESSTRQN

I10	LPLKGESSTRQNTMV
I11	KGESSTRQNTMVESS
I12	SSTRQNTMVESSDEE
I13	RQNTMVESSDEEDED
I14	TMVESSDEEDEDEGI
I15	ESSDEEDEDEGIVVR
I16	DEEDEDEGIVVRIDS
I17	DEDEGIVVRIDSPDK
I18	EGIVVRIDSPDKIVF
I19	VVRIDSPDKIVFRND
I20	VRIDSPDKIVFRNDL
I21	-
I22	-
I23	-
I24	-
I25	-
I26	-
I27	-
I28	-
I29	-
I30	-

Table S3. Oligonucleotides used for the generation of gene constructs and for qRT-PCR

SOS1_for	ATGACGACTGTAATCGACGC
SOS1_rev	TCATAGATCGTTCCTGAAAACG
SOS1C_EcoRI-Strep_for	TTTGAATTCATGTGGAGCCACCCACAGTTCGAAA AGTTTGTTCTACGC
SOS1_XbaI_rev	TTTTCTAGATCATAGATCGTTCCTGAAAACGATTTTACT
SOS1C_for-gateway	GGGGACAAGTTTGTACAAAAAAGCAGGCTTCATGTTTGTT CTACGCCTTCTTCG
SOS1_rev-gateway	GGGGACCACTTTGTACAAGAAAGCTGGGTCTAGATCGTTC CTGAAAACG
RTSOS1for	TTTGGAGCATTTGGAGCTGATAG
RTSOS1rev	TTTCTTTCTGATTTGGCTGAAACG
RTSOS1-N_for	TTTGGAATTCATGGCGATTTCTTTTTC
RTSOS1-N_rev	TTTGAAGGCGTAGAACAAATTGGG
PP2a_s	TAACGTGGCCAAAATGATGC
PP2a_as	GTTCTCCACAACCGCTTGGT
WRKY_fwd RT	GTGAAGATGAAGGGATGTC
WRKY_rev RT	CACAACCTTGGAATGTGC
FT_fwd RT	CCCTGCTACAACTGGAACAAC
FT_rev RT	CACCCTGGTGCATACACTG
14-3-3ω_for	TTTATGGCGTCTGGGCGTGAAG
14-3-3ω_rev	TTTTCACTGCTGTTCCTCGGTCG
14-3-3υ_for	TTTATGTCTTCTGATTCGTCCCGG
14-3-3υ_rev	TTTTCACTGCGAAGGTGGTGGTTG
14-3-3κ_for	TTTATGGCGACGACCTTAAGCAG
14-3-3κ_rev	TTTTCAGGCCTCATCCATCTGC
14-3-3λ_for	TTTATGGCGGCGACATTAGGCAG

14-3-3λ_rev	TTTTCAGGCCTCGTCCATCTGC
14-3-3ω_for-gateway	GGGGACAAGTTTGTACAAAAAAGCAGGCTTCATGGCGTCT GGGCGTGAAG
14-3-3ω_rev-gateway	GGGGACCACTTTGTACAAGAAAGCTGGGTCCTGCTGTTC CTCGGTC
14-3-3υ_for-gateway	GGGGACAAGTTTGTACAAAAAAGCAGGCTTCATGTCTT CTGATTCGTCCCGGGAAG
14-3-3υ_rev-gateway	GGGGACCACTTTGTACAAGAAAGCTGGGTCCTGCGAAGGT GGTGGTTG
14-3-3κ_for-gateway	GGGGACAAGTTTGTACAAAAAAGCAGGCTTCATGGCGACG ACCTTAAGCAGAGATC
14-3-3κ_rev-gateway	GGGGACCACTTTGTACAAGAAAGCTGGGTCGGCCTCA TCCATCTGC
14-3-3λ_for-gateway	GGGGACAAGTTTGTACAAAAAAGCAGGCTTCATGGCG GCGACATTAGGCAGAGAC
14-3-3λ_rev-gateway	GGGGACCACTTTGTACAAGAAAGCTGGGTCTCAGGCC TCGTCCATCTGCTCCTG
14-3-3ω_for_pET16b (NdeI)	TTTCATATGGCGTCTGGGCGTGAAG
14-3-3ω_rev_pET16b (BamHI)	TTTGGATCCTCACTGCTGTTCCTCG
SOS978f	GGGGACAAGTTTGTACAAAAAAGCAGGCTCTGATAGGACT CTACATCGAAGACCATCTTCGTTA
SOS3‘1146	GGGGACCACTTTGTACAAGAAAGCTGGGTCCTATCAT AGATCGTTCCTGAAAACGATTTT

Suppl. Information to:

Materials and Methods

Plant material and growth conditions

Wild type and mutant Arabidopsis (*Arabidopsis thaliana* (L.) Heynh., ecotype Columbia) plants were grown in a growth chamber at 21°C (day and night), 125 µE and short day conditions (10h light, 14h darkness). Plant growth was conducted on either soil, or in hydroponics or in sterile liquid culture. Experiments on soil were performed on standard fertilized soil (ED-73). After 3 weeks of growth under short-day conditions the plants were transferred to a plant chamber with 18 h of light (long-day conditions). Subsequently, soil was watered (tap water) with the same volume of either 150 mM NaCl solution or pure tap water every 3 d for a period of 2 weeks. Plants were grown for an additional 2 weeks before analyses were initiated. Growth in hydroponics was carried out in a nutrient medium as described in [1]. Experiments were performed with 5 week old plants, watered either with or without NaCl. Plant material from shoot and root tissue was used for ion and metabolite quantification and mRNA isolation.

For hydroponic experiments plants were cultivated in sterile liquid culture medium according to [2]. Liquid culture medium was replaced after 7 days with ±100 mM NaCl. Seeds used for sterile culture were surface sterilized in 5% sodium hypochloride and subsequently incubated for 2 d in the dark at 4 °C for stratification. The seedlings were harvested after 10 days and washed twice in deinonized water to remove external medium.

Generally, plant tissues were collected and immediately frozen in liquid nitrogen until use. Solute extraction from Arabidopsis tissues and spectroscopic quantification of sugars and starch were performed as described earlier [3].

Generation of *p35s::SOS1$^{c\text{-}term}$StrepII* plants

To create C-term overexpressor mutants the SOS1 amino acid sequence 446 to 1146 was cloned as a Strep-tagII-SOS1 C-terminus fusion construct and introduced into the plant genome via the floral dip method [4]. The Strep-tagII (WSHPQFEK) was introduced with the aim to quantify the recombinant SOS1-C-terminus protein via immuno-detection. It was added by the usage of the following oligonucleotides: SOS1C_Strep_for (5'-TTTGAATTCATGTGGAGCCACCCACAGTTCGAAAAGTTTGTTCTA CGC-3') and SOS1_XbaI_rev (5'-TTTTCTAGATCATAGATCGTTCCTGAAAACGATTTTACT-3'). We excised the mutated sequence by using *EcoR*I and *Xba*I and inserted it into the correspondingly prepared original pHannibal vector [5]. Then, the Strep-tagII-SOS1 C-terminus construct was cut by *Not*I and inserted into the correspondingly prepared original pART27 vector [6]. The fidelity of all constructs was confirmed by complete sequencing. Transformation of the pART27 vector harboring the *p35s::SOS1$^{c\text{-}term}$StrepII* construct was performed using *Agrobacterium tumefaciens,* strain GV3101. Positive transformants were isolated by Kanamycin selection.

Gene expression analysis

mRNA from Arabidopsis was extracted from frozen leaf and root material using the NucleoSpin® RNA Plant Kit (Machery-Nagel, Düren, Germany). Contaminating DNA was removed by DNase digestion. The

iScript™cDNA Synthesis Kit (Bio-Rad, Munich, Germany) was used for synthesis of cDNA. Quantitative real-time PCR analyses were performed using MyIQ.cycler and IQ SYBR Green supermix (Bio-Rad, Munich, Germany) with the following cycler conditions: 20 min at 50°C, 15 min at 95°C and 50 cycles of 15 s at 95°C, 25 s at 58°C and 40 s at 72°C. Data are calculated using the $2^{-\Delta\Delta Ct}$ formula [7] and either normalized to the phosphatase subunit *pp2a* (*At1g13320*) or to starting mRNA levels. Gene-specific oligonucleotides used for qRT-PCR are listed in Suppl. Table S3.

Interaction studies on *At*SOS1-C-terminus using Bimolecular Fluorescence Complementation (BiFC)

We verified of the interaction between the SOS1-C-terminus and the identified binding partners *in vivo* via the Bimolecular Fluorescence Complementation (BiFC) method. For this purpose we generated YFP^N and YFP^C fusion constructs using the GATEWAY™ specific destination vectors pUBC-cYFP-Dest, pUBC-nYFP-Dest, pUBN-cYFP-Dest and pUBN-nYFP-Dest [8]. The cDNA of the gene of interest was amplified by PCR using gene-specific primers harboring the *attB1* and *attB2* sites, then cloned via BP reaction into pDONRZEO (Invitrogen, Darmstadt, Germany) and via LR reaction into the specific destination vector (for primers used see Suppl. Table S3). Purified vector DNAs were introduced into Agrobacterium strain GV3101::PM90 which was subsequently used to transform epidermal cells of *Nicotiana benthamiana*. After an expression period of 3-5 days epidermal cells were analyzed with a Leica TCS SP5II confocal laser scanning microscope system.

Metabolite/ion extraction and quantifications

For isolation of sugars, proline and cations, plant material was ground under liquid N_2. Subsequently, one ml of deionized water was added to 100 mg tissue, the preparation was thoroughly mixed and kept for 15 min at 95°C. After centrifugation in an Eppendorf centrifuge (10 min, 13.000rpm, 4°C) the supernatant was used for ion chromatography quantifications. Quantification of cations was performed by using a Metrohm 761-IC Compact-System, equipped with a Metrosep-A-Supp1-250-Column (Metrohm, http://www.metrohm. com) followed by conductivity quantification at a flow rate of 1 ml min^{-1}, using 0.1 N HNO_3 and 1.7 mM dipicolinic acid as the mobile phase. Sugar quantification was performed by high pressure liquid chromatography on a Metrosep CARB1-150 column, using an 871-IC compact device (Metrohm) followed by amperometric quantification. NaOH (0.1 M) in double distilled water was used as the mobile phase with a flow rate of 1 ml min^{-1}. For proline quantification the amino acids and standards (20 mM each) were derivatized as described previously [9] using 6- aminoquinolyl-carbamyl (AQC; Watrex, http://www.watrex.com/). Measurements of the AQC amino acids were performed using a Dionex P680 HPLC system with an UV170U detector (Dionex, http://www.dionex.com/), and a column system consisting of a CC8/4 ND 100–5 C18–ec column and a 250/4 Nucledur 100–5 C18– ec column (Macherey-Nagel, Düren, Germany). A gradient comprising 100 mM sodium acetate and acetonitrile (0–15%) was used to separate the amino acids. The AQC amino acids were detected by fluorescence with excitation at 250 nm and emission at 395 nm.

Heterologous synthesis of *At*SOS1 C-terminus (aa978-1146) and *At*14-3-3 ω

To generate a N-terminal GST-tag fusion construct, the *At*SOS1 C-terminus(aa978-1146) coding sequence was amplified and inserted into a pDEST™15 plasmid (ThermoFisher Scientific). The pDEST™15 construct containing *At*SOS1 C-terminus (aa978-1146) was transformed into BL21-SI cells. The transformed bacterial cells were grown at 30°C in Luria-Bertani medium without NaCl. At an OD_{600} of 0.6 initiation of expression was

induced and cells were harvested 18 h post-induction by centrifugation in an Eppendorf centrifuge (6000rpm, 15 min, 4°C), resuspended in buffer medium (10 mM Tris-HCl, pH 7.4) and immediately frozen in liquid nitrogen. To avoid proteolytic degradation phenylmethane sulphonyl-fluoride (PMSF, 1 mM) and protease inhibitor cocktail (Roche, Penzberg, Germany) was added. Cell lysis was performed using a cell disrupter (Emulsiflex) at 5000-10000PSI. After centrifugation (10000x*g*, 15 min at 4°C), cell debris was removed and the supernatant was collected for the following purification of the soluble protein.

To generate an N-terminal (10xHis)-tag fusion construct, the *At*14-3-3 ω coding sequence was amplified and inserted into a pET16b plasmid (Novagen). The pET16b construct containing *At*14-3-3 ω was transformed into Rosetta(DE3) cells and transformed cells were grown at 37°C in Terrific Broth medium. At an OD_{600} of 0.6 initiation of expression was induced by addition of isopropyl-b-D-thiogalactopyranoside (IPTG, 1 mM). The cells were harvested 4 h post-induction by centrifugation (6000*x*rpm, 15 min, 4°C), re-suspended in buffer medium (50 mM Na_2HPO_4, 300 mM NaCl at pH 8.0) and immediately frozen in liquid N_2. To avoid proteolytic activity, phenylmethane sulphonyl-fluoride (PMSF, 1 mM) and protease inhibitor cocktail (Roche) was added. Cell lysis was supported by sonication. Via centrifugation (12500 rpm, 20 min, 4°C) cell debris were removed and the supernatant was collected for the following purification of the soluble protein.

Purification of *At*SOS1 C-terminus (aa978-1146) and *At*14-3-3 ω

All purification steps were carried out at 4°C. The supernatant containing the GST-tagged *At*SOS1 C-terminus (aa978-1146) construct was incubated with Glutathione Sepharose (Sigma-Aldrich) for 2 h. The suspension was transferred onto a chromatography column and washed with twice the sample volume with 10 mM PBST (Phosphate-Buffered Saline, 0,05% Tween20 at pH 7.4). Subsequently, recombinant *At*SOS1 C-terminus(aa978-1146) was eluted with 15 ml of elution buffer medium (50 mM Tris-HCl at pH 9.0, 7.5 mM reduced glutathione) and protein concentrations were quantified using the Pierce™ BCA protein assay kit (ThermoFisher Scientific).

The supernatant containing the (10xHis)-labeled *At*14-3-3 ω construct was incubated with NiNTA agarose (Qiagen, Düsseldorf, Germany) for 2 h. The Ni-NTA suspension was transferred onto a chromatography column, washed with 10 volumes of the Ni-NTA agarose bed volume with binding buffer medium (300 mM NaCl, 50 mM Na_2HPO_4, pH 8.0, 20 mM imidazole) and afterwards, with 6 column volumes of washing buffer medium (300 mM NaCl, 50 mM Na_2HPO_4, pH 8.0, 60 mM imidazole). Subsequently, recombinant *At*14-3-3 ω was eluted with 2 ml of elution buffer (300 mM NaCl, 50 mM Na_2HPO_4 at pH 8.0, 500 mM imidazole) and resulting protein concentrations were quantified.

Pull-down assay

The GST-tagged *At*SOS1 C-terminus(aa978-1146) peptide was transferred from elution buffer (50 mM Tris-HCl, pH 9.0, 7.5 mM reduced glutathione) to binding buffer medium (0.1 M MOPS, pH 7.5) by the use of an Amicon® Ultra Centrifugal filter (MerckMillipore, http://www.merckmillipore.com) and subsequently coupled to Affi-Gel10 matrix (Bio-Rad, http://www.bio-rad.com). Leaves of 5-week-old Arabidopsis plants were grounded in PBS medium, and the resulting extract was filtered prior to centrifugation. The supernatant was flushed over a chromatography column containing Affi-Gel10 affinity media to which the peptide had been coupled. The

column was then flushed successively with binding and washing buffer. Finally, the *At*SOS1 C-terminus (aa978-1146) and interacting proteins were eluted with elution buffer (PBS + 1% SDS), and proteins were precipitated by adding four volumes of ice-cold acetone, followed by centrifugation. The sediment was re-suspended in SDS–PAGE loading medium and proteins were separated by gel-electrophoresis After Coomassie Brilliant Blue R 250 staining the whole lane was excised and sliced into small horizontal pieces. Gel slices were analyzed by Mass Spectrometry. MS/MS spectra were searched against the Arabidopsis Information Resource (http:www.arabidopsis.org) database.

SDS-PAGE and immunostaining

SDS-PAGE was performed and separated proteins were stained with Coomassie Brilliant Blue R 250 or used for immunoblots. Immunodetection was carried out with a monoclonal anti-GST antibody (GE Healthcare, http://www.gehealthcare.com) in combination with a secondary horseradish peroxidase (HRP) anti-mouse IgG (http://www.sigmaaldrich.com). The apparent protein masses were estimated with Page RulerTM Prestained Protein Ladder (ThermoFisher Scientific).

Dot blot-analyses

Detection of the recombinant, Strep-taged SOS1 C-terminus protein in *p35s::SOS1$^{c\text{-}term}$StrepII* plants, was carried out using salt-stressed Arabidopsis root tissues. For this purpose, Arabidopsis Wt- and *p35s::SOS1$^{c\text{-}term}$StrepII* plants were cultivated in hydroponic culture for two weeks prior to exposure to 200 mM NaCl for 5 days. Collected plant material was ground under liquid N_2. Subsequently, 300 µl of 50 mM HEPES buffer, pH 7.6 (KOH) containing 1.5 mM PMSF was added to 300 mg tissue and the preparation was thoroughly mixed. After centrifugation in an Eppendorf centrifuge (10 min, 13000rpm, 4°C) the supernatant was transferred into a new reaction tube. In order to increase the protein concentration, an acetone precipitation was performed over night at -20°C. To this end, 4 volumes of cold acetone were added to 1 volume of sample. The obtained protein pellet was washed twice with 500 µl cold acetone the next day and re-suspended in 30 µl transparent SDS loading dye. After determination of the protein concentration, the precipitated samples were dropped on a cellulose membrane. Immunodetection was carried out with a monoclonal anti-Strep·Tag® antibody in combination with a secondary horseradish peroxidase (HRP) anti-mouse IgG. The apparent protein masses were estimated with Page RulerTM Prestained Protein Ladder (ThermoFisher Scientific). As a loading control, the blot was stained with Ponceau-S solution prior to immunoprinting.

Peptide-spot binding assay

15 amino acid long peptides, which cover the wild-type sequence of SOS1-C-terminus with an overlap of 12 aa were synthesized and immobilized on a cellulose membrane, derivatized with a polyethylene glycol spacer as described [10]. Membranes were activated in methanol for 4 min and then equilibrated in binding buffer (0.2 mM MOPS, 10 mM NaCl at pH 7.4) for 10 min. (10xHis)-labeled *At*14-3-3 ω was added and incubated at 4°C overnight. The membrane was blocked with TBS+2% milk powder and incubated with an anti-poly-Histidine antibody for 2 h, followed by incubation with the second anti-mouse antibody. The membrane was washed with TBS buffer three times for 10 min each, treated with ECL™, and subjected to luminescence imaging using an Odyssey® Fc Imaging System (LI-COR Biosciences, USA).

References

1. Conn, S.; Gilliham, M. Comparative physiology of elemental distributions in plants. *Ann. Bot.* **2010**, *105*, 1081-1102
2. Scheible, W.R.; Morcuende, R.; Czechowski, T.; Fritz, C.; Osuna, D.; Palacios-Rojas, N.; Schindelasch, D.; Thimm, O.; Udvardi, M.K.; Stitt, M. Genome-wide reprogramming of primary and secondary metabolism, protein synthesis, cellular growth processes, and the regulatory infrastructure of Arabidopsis in response to nitrogen. *Plant Physiol.* **2004**, *136*, 2483-2499.
3. Quick, W.P.; Neuhaus, H.E.; Feil, R.; Stitt, M. Fluoride leads to an increase of inorganic pyrophosphate and an inhibition of photosynthetic sucrose synthesis in spinach leaves. *Biochim. Biophys. Acta* **1989,** *973*, 263-271.
4. Clough, S.J.; Bent, A.F. Floral dip: a simplified method for Agrobacterium-mediated transformation of *Arabidopsis thaliana*. *Plant J.* **1998,** *16*, 735-743.
5. Wesley, S.V.; Helliwell, C.A.; Smith, S.A.; Wang, N.B.; Rouse, D.T.; Liu, Q.; Gooding, P.S.; Sing, S.P.; Abbott, P.; Stoutjesdijk, P.A., et al. Construct design for efficient, effective and high-throughput gene silencing in plants. *Plant J.* **2001,** *27*, 581-590.
6. Gleave; A.P. A versatile binary vector system with a T-DNA organisational structure conducive to efficient integration of cloned DNA into the plant genome. *Plant Mol. Biol.* **1992,** *20*, 1203-1210.
7. Livak, K.J.; Schmittgen, T.D. Analysis of relative gene expression data using real-time quantitative PCR and the $2^{-\Delta\Delta CT}$ method. *Methods* **2001**, *25*, 402-408.
8. Grefen, C.; Chen, Z.; Honsbein, A., Donald, N.; Hills, A., Blatt M.R.. A novel motif essential for SNARE interaction with the $K^{(+)}$ channel KC1 and channel gating in Arabidopsis. *Plant Cell* **2010,** *22*, 3076-3092.
9. Rolletschek, H.; Hajirezaei, M.R.; Wobus, U.; Weber, H. Antisense-inhibition of ADP-glucose pyrophosphorylase in *Vicia narbonensis* seeds increases soluble sugars and leads to higher water and nitrogen uptake. *Planta* **2002,** *214*, 954-964.
10. Hilpert, K.; Winklere, D.F.; Hancock, R.E. Peptide arrays on cellulose support: SPOT synthesis, a time and cost efficient method for synthesis of large numbers of peptides in a parallel and addressable fashion. *Nat. Protoc.* **2007**,*2*, 1333-1349
